# Dynamic association of antimicrobial resistance in urinary isolates of *Escherichia coli* and *Klebsiella pneumoniae* between primary care and hospital settings in the Netherlands (2008–2020): a population-based study

**DOI:** 10.1016/j.lanepe.2024.101197

**Published:** 2025-01-03

**Authors:** Evelyn Pamela Martínez, Annelies Verbon, Annelot F. Schoffelen, Wieke Altorf-van der Kuil, Joost van Rosmalen, ISIS-AR study group, James W.T. Cohen Stuart, Damian C. Melles, Karin van Dijk, Ali Alzubaidy, Maarten Scholing, Sacha D. Kuil, Gert J. Blaauw, Wieke Altorf - van der Kuil, Saskia M. Bierman, Sabine C. de Greeff, Sonja R. Groenendijk, Rudy Hertroys, Jos C.M. Monen, Daan W. Notermans, Jan Polman, Wouter J. van den Reek, Caroline Schneeberger-van der Linden, Annelot F. Schoffelen, Feline Velthuis, Cornelia C.H. Wielders, Britt J. de Wit, Rony E. Zoetigheid, Wouter van den Bijllaardt, Elise M. Kraan, Michiel B. Haeseker, Júlia M. da Silva, Eefje de Jong, Boulos Maraha, Arjanne J. van Griethuysen, Bastiaan B. Wintermans, Marijke J.C.A. van Trijp, Anouk E. Muller, Man-Chi Wong, Alewijn Ott, Erik Bathoorn, Mariëtte Lokate, Jan Sinnige, Damian C. Melles, Nienke Plantinga, Nicole H.M. Renders, Julia W. Dorigo-Zetsma, Leendert J. Bakker, Wim Ang, Karola Waar, Martha T. van der Beek, Maurine A. Leversteijn-van Hall, Suzan P. van Mens, Erik Schaftenaar, Marrigje H. Nabuurs-Franssen, Ianthe Maat, Patrick D.J. Sturm, Bram M.W. Diederen, Lonneke G.M. Bode, David S.Y. Ong, Michiel van Rijn, Sander Dinant, Martijn den Reijer, Dick W. van Dam, Els I.G.B. de Brauwer, Robbert G. Bentvelsen, Anton G.M. Buiting, Anne L.M. Vlek, Melanie de Graaf, Annet Troelstra, Arjan R. Jansz, Maurits P.A. van Meer, Janneke de Vries, Julian D. Machiels

**Affiliations:** a Facultad de Medicina Veterinaria y Zootecnia, Universidad Central del Ecuador, Quito, Ecuador; b Department of Medical Microbiology and Infectious Diseases, Erasmus MC, University Medical Centre, Rotterdam, the Netherlands; c Department of Infectious Diseases, Division of Internal Medicine and Dermatology, University Medical Center Utrecht, Utrecht University, the Netherlands; d Centre for Infectious Diseases, Epidemiology and Surveillance, Dutch National Institute for Public Health and the Environment (RIVM), Bilthoven, the Netherlands; e Department of Biostatistics, Erasmus MC, University Medical Centre, Rotterdam, the Netherlands; f Department of Epidemiology, Erasmus MC, University Medical Centre, Rotterdam, the Netherlands; g Julius Center for Health Sciences and Primary Care, University Medical Center Utrecht, Utrecht University, Utrecht, the Netherlands

**Keywords:** Antimicrobial resistance, General practitioners, Health care settings, Granger causality, Multiple time series

## Abstract

**Background:**

It is unclear whether changes in antimicrobial resistance (AMR) in primary care influence AMR in hospital settings. Therefore, we investigated the dynamic association of AMR between primary care and hospitals.

**Methods:**

We studied resistance percentages of *Escherichia coli* and *Klebsiella pneumoniae* isolates to co-amoxiclav, ciprofloxacin, fosfomycin, nitrofurantoin and trimethoprim submitted by primary care, hospital outpatient and hospital inpatient settings to the Dutch National AMR surveillance network (ISIS-AR) from 2008 to 2020. For each bacterium–antibiotic combination, we first conducted multivariable logistic regressions to calculate AMR odds ratios (ORs) by month and healthcare setting, adjusted for patient-related factors and a time term. Second, multiple time series analysis was done using vector autoregressive models including the (log) ORs for each bacterium–antibiotic combination. Models were interpreted by impulse response functions and Granger-causality tests.

**Findings:**

The main AMR association was unidirectional from primary care to hospital settings with Granger-causality p-values between <0.0001 and 0.029. Depending on the bacterium–antibiotic combination, a 1% increase of AMR in *E. coli* and *K. pneumoniae* in primary care leads to an increase of AMR in hospital settings ranging from 0.10% to 0.40%. For ciprofloxacin resistance in *K. pneumoniae,* we found significant bidirectional associations between all healthcare settings with Granger-causality p-values between <0.0001 and 0.0075.

**Interpretation:**

For the majority of bacterium–antibiotic combinations, the main AMR association was from primary care to hospital settings. These results underscore the importance of antibiotic stewardship at the community level.

**Funding:**

ISIS-AR is supported by the Ministry of Health, Welfare and Sport of the Netherlands and the first author by the 10.13039/100019134Central University of Ecuador to follow a PhD program in Erasmus MC.

**Research in context**

**Evidence before this study**

We searched for relevant studies in PubMed with the terms (“antimicrobial resistance” OR “antibiotic resistance”) AND (“outpatient” OR “primary care” OR “community”) AND (“hospital” OR “inpatient” OR “secondary”) AND (“time series”), on January 10th, 2021. We limited our search within the title and abstract of published studies independently of the type of study, and we used no date and language restrictions. To the results of this search, we added published relevant studies found by direct search in Google Scholar. The search returned 30 studies, from which we excluded seven studies focused on evaluating antibiotic stewardship programs at primary care or/and hospital level. We also excluded reviews or systematic reviews, three in total. Nine studies used time series analysis to evaluate the association between antibiotic use and antimicrobial resistance (AMR) in either primary care or hospitals in the United States, France, Spain and Switzerland. Most available studies focus on common bacteria such as *Escherichia coli,* and resistance to commonly used antibiotics in both primary care and hospitals, and used advanced time series analysis techniques such as ARIMA models, VAR models, and transfer functions. One study used time series analysis to describe methicillin-resistance *Staphylococcus aureus* in respect to specimen source, patient location and environmental temperature. Two studies used time series analysis to describe trends of antibiotic use in primary care. Although there is evidence from previous studies exploring AMR and AMU using time series analysis, none of them focused on describing the association of AMR between primary care and hospitals.

In addition, we searched for resistance data from the Dutch National AMR Surveillance Network, the Infectious Disease Surveillance Information System for Antibiotic Resistance (ISIS-AR), on June 9th, 2021. We focused on data of resistance in two bacteria commonly causing urinary tract infections (UTIs), *E. coli* and *Klebsiella pneumoniae*, against antibiotics UTIs such as nitrofurantoin, fosfomycin, trimethoprim, ciprofloxacin and the combination of amoxicillin and clavulanic acid.

**Added value of this study**

We wanted to determine if past changes of AMR in primary care could predict future changes of AMR in hospital settings in the Netherlands using time series analysis. By applying VAR models, it was possible to determine how an increase of AMR in *E. coli* and *K. pneumoniae* in primary care could lead to an increase of AMR in hospitals in the future. Our results also showed that the magnitude of the increase in AMR in hospitals after an increase of AMR in primary care depends on the bacterium–antibiotic combination. Furthermore, it was observed that the association of AMR was bidirectional between primary care and hospital for ciprofloxacin resistance in both bacteria, probably due to its usage across healthcare settings. Our findings have added to the available evidence regarding the dynamic phenomenon of AMR emergence across healthcare settings.

**Implications of all the available evidence**

This study reveals that AMR in primary care and hospitals in the Netherlands is an interconnected system, in which any change in AMR in one health care setting could lead to changes in AMR in other health care setting. The fact that the main AMR association was from primary care to hospitals highlights the importance of antibiotic stewardship at the community level, as antibiotic use is still considered the main driver of AMR. Further studies are needed to elucidate the association of AMR in different health care settings and for other antibiotics.

## Introduction

Use of antibiotics in primary care has been associated with higher levels of antimicrobial resistance (AMR) in primary care itself and in hospitals.^1^^,^^2^ A study in the United States found that high prescription rates of penicillins in primary care were associated with *E. coli* being resistant to penicillins in both outpatients and inpatients, with a time lag of 1–2 months.^3^ Similarly, studies in France and Switzerland showed that ciprofloxacin-resistant *E. coli* from hospitals was linked to high consumption of fluoroquinolones within hospitals and in primary care, with a time lag ranging from 1 to 12 months.^2^^,^^4^ These studies have shown that resistant bacteria circulating in the community may be a source of AMR in hospitals. However, previous studies have focused on the association between antibiotic use and AMR, and not specifically on the association of AMR between primary care and hospital settings. Knowing the presence and the direction of these associations would guide strategies for reducing AMR.

Across Europe, wide differences exist in the total amount of antibiotic use and resistance percentages. In 2022, the Netherlands was ranked as the country with the lowest antibiotic use (community and hospitals) in Europe with a mean total use of 9.1 DDD per 1000 inhabitants per day (DID) compared to countries such as Belgium (20.4 DID), France (24.3 DID) and Cyprus (33.5 DID).^5^ In concordance, resistance percentages in primary care and hospital settings in the Netherlands are also relatively low compared to other European countries.^6^ Urinary tract infection (UTI) is the most frequent reason for antibiotic use in primary care,^7^ with approximately 30 million packages of broad-spectrum antibiotics prescribed to treat UTIs across Europe in 2019.^8^ UTIs affect approximately 150 million people per year, predominantly women.^8^ In the Netherlands, in 2022, there were 149 visits for UTIs in primary care per 1000 registered patients.^9^ In hospital settings 19% of 15,000 reported hospital-associated infections were UTIs in European countries.^10^ *E. coli* and *K. pneumoniae* are the most common bacteria causing UTIs, and AMR in these bacteria has been increasing in all healthcare settings.^6^^,^^11^, ^12^, ^13^ This makes *E. coli* and *K. pneumoniae* causing UTIs suitable micro-organisms to study the dynamic of AMR between primary care and hospital settings, and to show whether AMR changes in one healthcare setting could influence AMR levels in other settings.

Considering that in the Netherlands approximately 80% of antibiotics are prescribed in primary care,^14^ we hypothesized that past changes of AMR in primary care could predict future changes of AMR in hospital settings. To test this hypothesis, we investigated the temporal associations of AMR between healthcare settings for the most common antibiotics used to treat uncomplicated and complicated UTIs in the Netherlands using multiple time series analysis.

## Methods

### Antimicrobial resistance data

For this population-based study, AMR data from January 2008 to December 2020 were obtained from the Dutch National AMR Surveillance Network, the Infectious Disease Surveillance Information System for Antibiotic Resistance (ISIS-AR), of which full details have been published elsewhere.^15^ In brief, ISIS-AR routinely collects antimicrobial susceptibility testing (AST) data of all positive cultures from medical routine diagnostics in participating clinical microbiology laboratories in the Netherlands, serving hospitals, general practices and long-term care facilities.^15^ Furthermore, for each isolate, some epidemiological information about the patient is collected, including year and month of birth, sex and type of healthcare setting and department where the sample was taken. Patient identification in ISIS-AR is based on a pseudonymization of the patient number of the laboratory. Consequently, follow-up samples of the same patient can be recognized within one laboratory, but not between laboratories. Within ISIS-AR it is possible to distinguish whether a sample was taken because of symptoms of infection (diagnostic isolates) or for screening purposes (screening isolates). However, no data on the clinical source of infection are collected. Therefore, for the current analysis, we assumed that diagnostic urinary samples were taken because of a urinary tract infection.

In 2020, 46 laboratories (out of a total of 51 laboratories in the Netherlands) were connected voluntarily to the ISIS-AR surveillance network. To avoid bias in the AMR time trends, we studied AST results only from 23 out of 46 laboratories that continuously provided data throughout the whole study period. We selected AST results of diagnostic *E. coli* and *K. pneumoniae* urine isolates against five antibiotics: nitrofurantoin (*E. coli* only), fosfomycin and trimethoprim used in primary care for uncomplicated UTIs, and ciprofloxacin and the combination of amoxicillin and clavulanic acid (co-amoxiclav) used for complicated UTIs in primary care and as empirical treatment in hospitals.^16^^,^^17^ AMR was determined from the first isolate per patient per year. To avoid bias in resistance percentages due to selective testing, for each laboratory, data on a specific bacterium–antibiotic combination was included only if in each separate year more than 50% of the available isolates of that bacterium were tested for the antibiotic of interest, and more than 80% of the MIC values that could be reinterpreted using EUCAST breakpoints version 10.0 (2020) were provided for the antibiotic of interest. With this selection process, 16%–30% of the resistance data were missing, depending on the specific bacterium–antibiotic combination. No missing values were present in the variables age and location (i.e., provinces), and the variable sex was missing in only 221 isolates.

Because in the years before 2014 many laboratories tested fosfomycin susceptibility only for a selection of isolates, we included data from 2014 to 2020 only for this antibiotic to avoid overestimation of AMR levels and to increase the power of the statistical analysis. For co-amoxiclav, a clear structural break (i.e., a sudden change at a point in time) was present in 2016 as a result of a switch in 2016 from CLSI (fixed ratio 2:1 of co-amoxiclav) to EUCAST (fixed 2 mg/L clavulanate concentration) in the automated susceptibility testing systems used in Dutch laboratories.^6^ This change led to higher resistance percentages in both *E. coli* and *K. pneumoniae* since 2016. Because a structural break in a time series can affect parameter estimates leading to wrong interpretation when performing a time series model, we used the data from January 2008 to December 2015.

### Calculation of antimicrobial resistance time series

AMR time series were constructed using the resistance percentages in *E. coli* and *K. pneumoniae* stratified by healthcare setting and antibiotic type. Resistance percentages for each species were calculated as the percentage of resistant isolates among the total number of tested isolates to a specific antibiotic per month and year.^6^^,^^12^ Healthcare settings were defined in line with the Dutch AMR national reports (NethMap) as: 1) Primary care: AMR data of patients exclusively from general practitioners (excluding urologists, gynecologists, urologists and pediatricians); 2) Hospital outpatient: AMR data only from outpatient departments; and 3) Hospital inpatient: AMR data from patients admitted to hospitals, excluding intensive care units (ICU).^6^ ICU patients were excluded since this population was too small to draw conclusions and because approximately 60%–80% of UTIs in ICU patients are associated with urinary catheters, making the diagnosis of UTI difficult in this setting.^6^^,^^18^ Additionally, in the Netherlands all ICU patients are prescribed selective bowel decontamination (Ceftriaxon), which influences resistance percentages.

For *E. coli*, AMR time series were constructed for all three healthcare settings for ciprofloxacin, nitrofurantoin, trimethoprim, fosfomycin, and co-amoxiclav, in total 15 time series. For *K. pneumoniae,* time series were constructed for the same antibiotics, except for nitrofurantoin, in total 12 time series.

### Data analysis

We used version 4.1.1 of the statistical software R using the packages *tseries*, *ggplot2*, *urca*, and *vars*. A two-step analysis was used to determine the dynamic association of AMR between primary care, hospital outpatient and hospital inpatient settings based on obtained data from ISIS-AR.

The aim of the first step was to reduce the variation of resistance percentages due to patient characteristics. Multivariable logistic regression analysis was used to adjust monthly AMR time series for patient-related factors. The outcome in these models was the antibiotic susceptibility pattern as a binary variable. Only for ciprofloxacin antibiotic there was an intermediate category that was recoded as susceptible. Available explanatory variables were sex, patient location (i.e., region where the patient lived at the moment of urine sampling, see Supplementary Fig. S1), age in years at the moment of urine sampling and a categorical variable *time*, which indicated the year and the month. The reference group of *time* was the month January of the first available year, which was 2008 for all antibiotics except of fosfomycin, which had data from 2014 onwards. The main results of the logistic regression analyses were the estimated odds ratios (ORs) for each month of the entire study period, which describe the temporal changes in AMR adjusted for patient-related factors, and which served as input for the second step of the analysis.

As second step, multiple time series analysis was performed using vector autoregressive (VAR) models. VAR models are traditionally used in econometrics, but they have also been applied in health science research, as a flexible approach for capturing the dynamic associations occurring between multiple time series, including reciprocal associations.^19^, ^20^, ^21^ In a VAR, a number of time series can be included as endogenous variables (which are changed or determined by their relationship with other variables in the model) to form a “system”. Inside the system, different equations are estimated by ordinary least squares (OLS) to explain the dynamic between the endogenous variables by relating the current observations of a variable to past observations of itself and the past observations of the other variables inside the system. Each equation includes an error term to capture variation in the outcome that is not explained by the model.^19^^,^^22^

We formed a VAR system using three endogenous variables corresponding to the log-transformed ORs per month per year calculated in step 1, representing AMR in the primary care, hospital outpatient and hospital inpatient settings. The error terms in the VAR model capture the uncertainty in the estimated log ORs that arises from the limited number of isolates per month of the study period. In total, five VAR models were built for *E. coli* and four for *K. pneumoniae,* corresponding to each bacterium–antibiotic combination. The multiple time series analysis was performed in four steps.^19^^,^^21^^,^^23^ First, we assessed whether each endogenous variable was stationary by analyzing the presence of a unit root and a trend using the Augmented Dickey–Fuller test. If a variable showed a trend, a trend component was considered in the VAR model (Supplementary Table S1). Second, the optimum lag length of each VAR was estimated using the Akaike Information Criterion, setting up a maximum of 6-month lags (Supplementary Table S2). Third, VAR models were built with the endogenous variables in levels (i.e., without differentiation), with the suggested number of lags, a constant term, a trend term when necessary, and centered seasonal dummies (Supplementary Table S3).^23^ Finally, diagnostic checks of each VAR system were done. Residuals were examined for normality, equal variance, and serial correlation using the Jarque–Bera test, the autoregressive conditional heteroscedasticity test, and the Portmanteau test, respectively (Supplementary Table S4). VAR model stability was visually assessed with the Ordinary Least Squares Cumulative Sum (OLS-CUSUM) test (Supplementary Fig. S2).^23^

Since our research question was whether changes in AMR in one setting led to changes of AMR in another setting, we interpreted VAR models using Granger-causality tests and impulse response functions (IRFs).^19^, ^20^, ^21^ Granger-causality tests can answer this question by analyzing the flow of the temporal association (unidirectional or bidirectional) between AMR in primary care and hospital settings. This test is based on the concept of Granger causality,^24^ where a time series “x” Granger-causes another time series “y” if the past values of “x” help to predict future values of “y” in a better way than the past values of “y” itself. Thus, Granger causality provides information about forecasting ability of one time series to another time series, but this test does not provide insight into a true causal relationship between variables.^24^ Since we had a VAR system with three variables, we tested Granger causality coming from one healthcare setting to the other two at the same time (pairwise analysis was not available in the *vars* R package). For instance, under the null hypothesis it was tested that AMR in primary care does not Granger-cause AMR in either hospital outpatient or hospital inpatient settings.

IRFs were used to predict how a change of AMR in one healthcare setting affects AMR in the other healthcare settings. For this, an imaginary impulse or a “shock” was applied in the model in one healthcare care setting and then the effects of this impulse in the other healthcare settings were calculated over a specified time horizon into the future.^21^ The IRFs were calculated for a small shock that consisted of an immediate 1% relative increase in AMR in one healthcare setting. The dynamic response on the other healthcare settings was traced out up to a horizon of 12 months. For interpretation, the response to that shock was expressed as a percentage (e.g., a 1% relative shock would represent an increase of AMR from 30% to 30.3%). The IRFs are shown in graphs and were computed as non-cumulative and non-orthogonal. Confidence intervals were obtained by bootstrapping with 1000 runs.^21^^,^^23^

### Role of the funding source

Funding sources did not have any involvement in the study design, data collection, data analysis and the interpretation of the data or writing of the manuscript. None of the authors have been paid by pharmaceutical companies or other agencies to write this manuscript. The authors were not precluded from accessing data in the study and all accept responsibility to submit for publication.

## Results

The majority of selected isolates was from women (70–79%, depending on the healthcare setting), and from patients aged over 65 years (47–69%). The place of residence of the patient at the moment of sampling was well distributed over the country with a slight emphasis on the West Region (approximately 42%) that included Utrecht, Noord-Holland, Zuid-Holland and Zeeland provinces. The highest resistance percentage in *E. coli* and *K. pneumoniae* were found in hospital outpatients. In *E. coli*, the highest resistance percentage was for trimethoprim (>25%), and the lowest was for fosfomycin (<2%) in all healthcare settings. In *K. pneumoniae*, high resistance percentage were found for trimethoprim (>22%) and fosfomycin (>23%), especially in primary care and hospital outpatients (Table 1).

**Table 1:** Summary of resistance isolates according to population characteristics by healthcare setting in the Netherlands from January 2008 to December 2020.

Variables	Primary care	Hospital outpatient	Hospital inpatient
Age in years, number of patients (%)			
0–4	30,189 (4)	7570 (4)	8748 (4)
5–18	65,116 (9)	11,774 (6)	3979 (2)
19–64	295,535 (40)	69,833 (34)	53,092 (25)
>65	347,390 (47)	115,329 (56)	146,799 (69)
Sex, number of patients (%)			
Female	581,900 (79)	143,006 (70)	149,930 (71)
Male	156,127 (21)	61,497 (30)	62,688 (29)
Region, number of patients (%)			
East	135,844 (19)	32,908 (16)	32,427 (15)
North	184,068 (25)	37,933 (19)	44,821 (21)
South	100,714 (14)	48,740 (24)	52,196 (25)
West	308,285 (42)	82,990 (41)	82,074 (39)
Antibiotics tested in *E. coli*, number of isolates (% resistant)			
Ciprofloxacin	577,960 (10)	146,916 (18)	160,450 (15)
Co-amoxiclav	359,808 (15)	962,59 (19)	97,707 (20)
Fosfomycin	367,040 (1)	71,742 (2)	71,591 (1)
Nitrofurantoin	630,325 (2)	142,122 (3)	148,060 (2)
Trimethoprim	629,518 (26)	149,955 (31)	157,404 (28)
Antibiotics tested in *K. pneumoniae*, number of isolates (% resistant)			
Ciprofloxacin	65,574 (12)	25,162 (12)	27,079 (11)
Co-amoxiclav	36,797 (8)	14,913 (10)	15,288 (12)
Fosfomycin	45,409 (31)	13,554 (28)	12,996 (23)
Trimethoprim	71,436 (23)	26,020 (24)	26,682 (20)

Resistance (%) was calculated by dividing the number of resistant isolates by the number of isolates tested for a specific antibiotic. The study period for ciprofloxacin, nitrofurantoin and trimethoprim was from January 2008 to December 2020; for co-amoxiclav it was from January 2008 to December 2015 and for fosfomycin from January 2014 to December 2020. Regions were defined according to the provinces in the Netherlands as follows: East (Overijssel, Gelderland, Flevoland); North (Groningen, Friesland, Drenthe); South (Noord-Brabant, Limburg); West (Utrecht, Noord-Holland, Zuid-Holland, Zeeland).

### Time trends of antimicrobial resistance

**Fig. 1:**
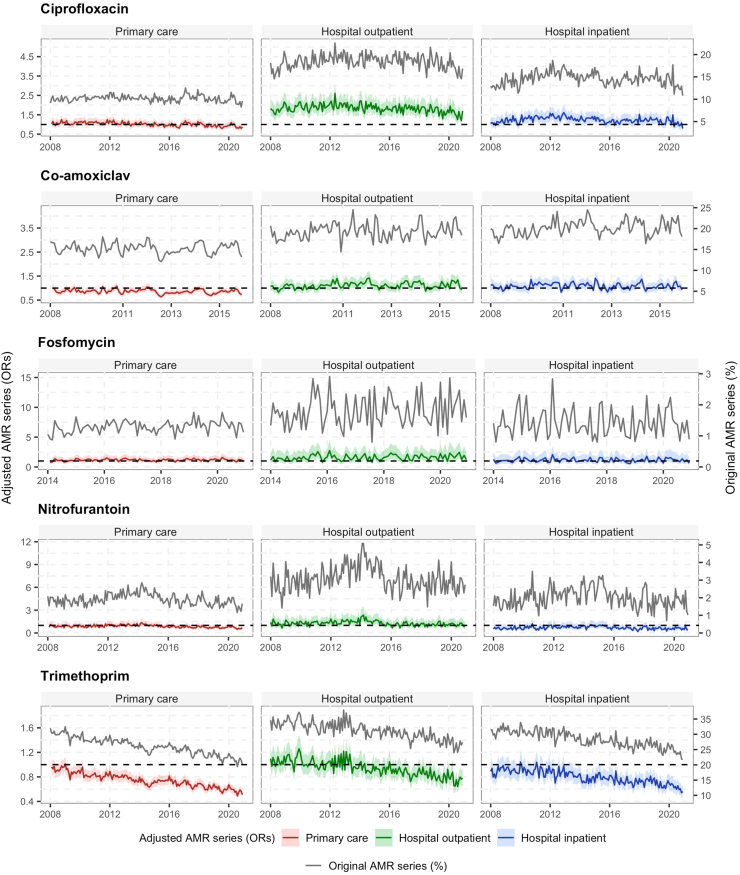
Antimicrobial resistance time trends in *E. coli* by healthcare settings and antibiotic type in the Netherlands. AMR series for ciprofloxacin, nitrofurantoin and trimethoprim are from 2008 to 2020. AMR series for co-amoxiclav are from 2008 to 2015, while AMR series for fosfomycin are from 2014 to 2020. Adjusted time series (OR of Time) with their 95% CIs (shadows) were calculated by multiple logistic regression analysis adjusted for patient-related factors and calendar time (left y-axis). As reference, the horizontal dotted line shows an OR = 1. Original time series (%) were calculated as the proportion of resistant isolates among the total number of tested isolates for each antibiotic of interest (right y-axis).

**Fig. 2:**
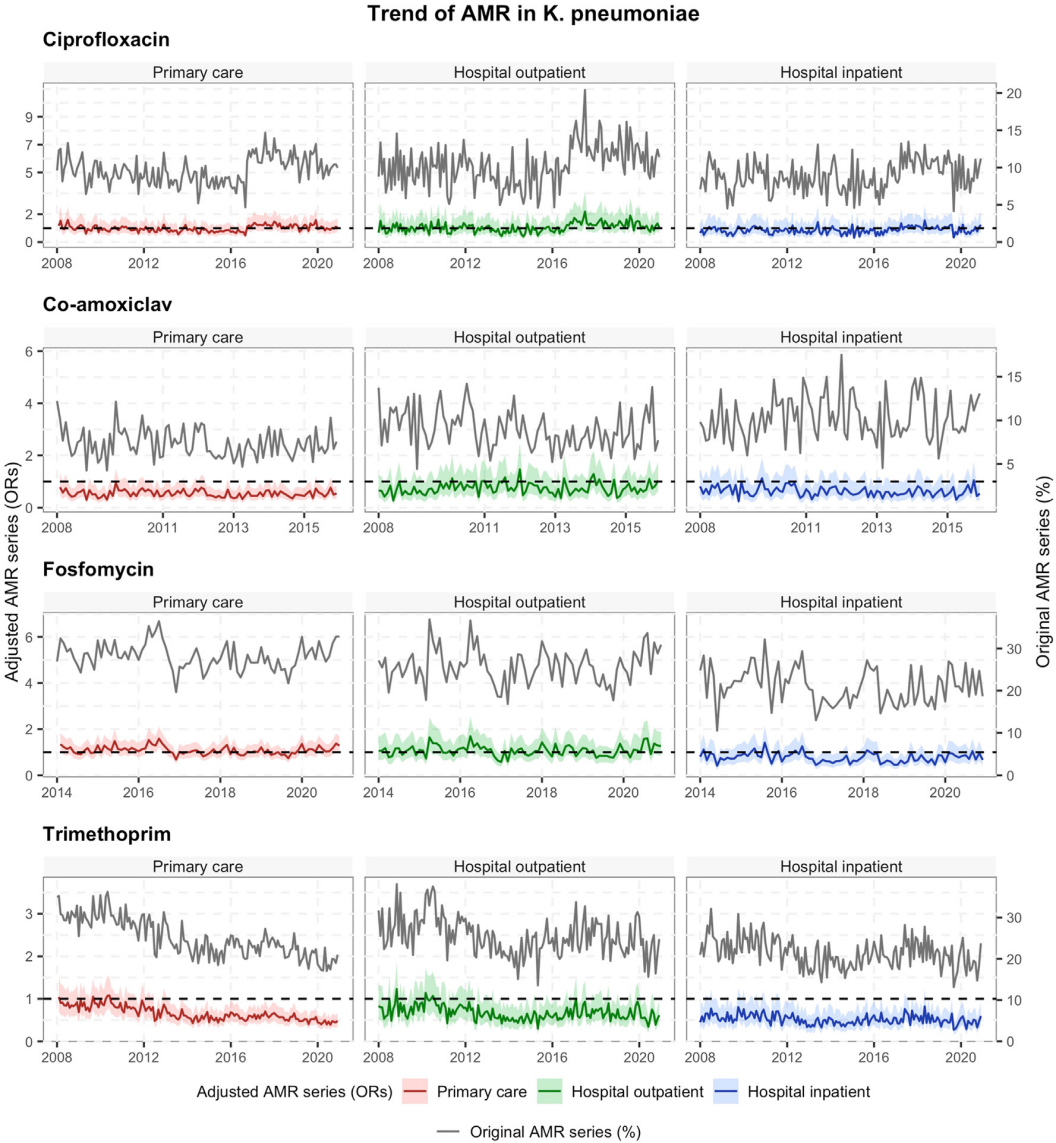
Antimicrobial resistance time trends in *K. pneumoniae* by healthcare settings and antibiotic type in the Netherlands. AMR series for ciprofloxacin, nitrofurantoin and trimethoprim are from 2008 to 2020. AMR series for co-amoxiclav are from 2008 to 2015, while AMR series for fosfomycin are from 2014 to 2020. Adjusted time series (OR of Time) with their 95% CIs (shadows) were calculated by multiple logistic regression analysis adjusted for patient related factors and calendar time (left y-axis). As reference, the horizontal dotted line shows an OR = 1. Original time series (%) were calculated as the proportion of resistant isolates among the total number of tested isolates for each antibiotic of interest (right y-axis).

Table 2 shows the relative changes in resistance percentages in *E. coli* and *K. pneumoniae*. From 2008 to 2020, ciprofloxacin resistance in *E. coli* decreased by 2.6% in primary care and by 1.3% in hospital outpatient settings, and increased by 1.8% in hospital inpatient setting. In *K. pneumoniae,* ciprofloxacin resistance decreased by 5.8% in primary care and by 4.0% in hospital inpatient setting. Nitrofurantoin resistance in *E. coli* increased in hospitals (9.4% in outpatients and 5.5% in inpatients), but decreased by 10.6% in primary care. Trimethoprim resistance in *E. coli* and *K. pneumoniae* showed a clear decreasing trend in all healthcare settings (Fig. 1, Fig. 2).

**Table 2:** Relative change of the percentage of resistance of *Escherichia coli* and *Klebsiella pneumoniae* from start to end of the study period by healthcare setting and antibiotic type in the Netherlands.

Antibiotic type	Relative change in resistance (%) from start to end of study period								
	Primary care			Hospital outpatient			Hospital inpatient		
	Start	End	Relative change (%)	Start	End	Relative change (%)	Start	End	Relative change (%)
*E. coli*									
Ciprofloxacin	9.9	9.7	−2.6	17.1	16.8	−1.3	12.9	13.1	1.8
Co-amoxiclav	15.5	15.8	2.1	18.4	20.2	9.8	19.4	21.1	9.1
Fosfomycin	1.1	1.3	20.2	1.5	2.0	27.3	1.4	1.3	−3.5
Nitrofurantoin	2.0	1.8	−10.7	2.8	3.0	9.4	1.8	1.9	5.5
Trimethoprim	30.3	21.5	−29.0	33.2	26.4	−20.4	30.9	24.1	−22.2
*K. pneumoniae*									
Ciprofloxacin	12.6	11.8	−5.8	11.6	13.2	13.4	11.1	10.6	−4.0
Co-amoxiclav	8.9	8.3	−7.3	11.6	10.0	−13.5	10.2	12.1	17.9
Fosfomycin	30.6	32.9	7.3	27.7	30.6	10.4	23.4	23.9	2.1
Trimethoprim	29.3	17.8	−39.2	26.5	21.4	−19.2	22.7	18.5	−18.5

Relative changes were calculated as the ratio of the difference between the end and start resistance percentage divided by the start resistance percentage. The study period for ciprofloxacin, nitrofurantoin and trimethoprim was from January 2008 to December 2020; for co-amoxiclav it was from January 2008 to December 2015 and for fosfomycin from January 2014 to December 2020.

From 2008 to 2015, co-amoxiclav resistance in *E. coli* increased by 2.1% in primary care and approximately by 9% in hospital settings. In *K. pneumoniae*, co-amoxiclav resistance increased by 17.9% in hospital inpatients, but it decreased in primary care and hospital outpatient setting by approximately 10%. From 2014 to 2020, fosfomycin resistance increased in all healthcare settings for *K. pneumoniae,* while in *E. coli*, fosfomycin resistance increased by 20.2% in primary care (from 1.1% to 1.3%) and by 27.3% in the hospital outpatient setting (from 1.5% to 2.0%), but not in the hospital inpatient setting (Fig. 1, Fig. 2).

### Temporal association of antimicrobial resistance between primary care and hospitals

VAR model coefficients showing the dynamic association of AMR in *E. coli* and *K. pneumoniae* between primary care and hospitals are showing in Supplementary Table S5.

For the majority of bacterium–antibiotic combinations, the main finding of Granger causality was from primary care to either hospital setting, meaning that AMR in hospital settings can be predicted by AMR in primary care (Table 3). Another relevant direction of Granger causality was from primary care and the hospital outpatient setting taken together to the hospital inpatient setting, notably for ciprofloxacin and fosfomycin resistance in *E. coli* (p = 0.00028 and p = 0.0034, respectively), and for ciprofloxacin and trimethoprim resistance in *K. pneumoniae* (p = 0.00072 and p = 0.0019, respectively) (Table 3).

**Table 3:** p-values for Granger causality of antimicrobial resistance in *E. coli* and *K. pneumoniae* between primary care, hospital outpatient and hospital inpatient settings in the Netherlands.

Antibiotic type	Granger causality flow					
	PC ⟶ HO and HI	HO ⟶ PC and HI	HI ⟶ PC and HO	PC and HO ⟶ HI	PC and HI ⟶ HO	HO and HI ⟶ PC
*E. coli*						
Ciprofloxacin	0.0013	0.022	0.073	0.00028	0.00023	0.64
Co-amoxiclav	0.36	0.70	0.19	0.54	0.12	0.10
Fosfomycin	0.016	0.41	0.92	0.0034	0.46	0.95
Nitrofurantoin	0.010	0.055	0.15	0.0081	0.00021	0.038
Trimethoprim	0.029	0.17	0.40	0.074	0.031	0.65
*K. pneumoniae*						
Ciprofloxacin	<0.0001	0.0075	0.00078	0.00072	<0.0001	0.00064
Co-amoxiclav	0.017	0.15	0.052	0.098	0.12	0.0059
Fosfomycin	0.10	0.16	0.0051	0.21	0.0016	0.0012
Trimethoprim	0.0032	0.017	0.40	0.0019	0.089	0.011

PC = primary care, HO = hospital outpatient, HI = hospital inpatient. Arrows show the direction flows of the Granger-causality between healthcare settings. A p-value < 0.05 means that the variable left of the arrow significantly “Granger causes” (i.e., can be used to predict) the variables on the right-hand side of the arrow. For example, AMR in PC granger causes AMR in HO and HI.

In *E. coli*, no significant Granger causality was found for co-amoxiclav (p > 0.36). In *K. pneumoniae*, the main direction of Granger causality for fosfomycin was from hospital settings to primary care (p = 0.0010), whereas for ciprofloxacin, there were significant bidirectional associations in all possible ways among healthcare settings (p < 0.0075) (Table 3).

**Fig. 3:**
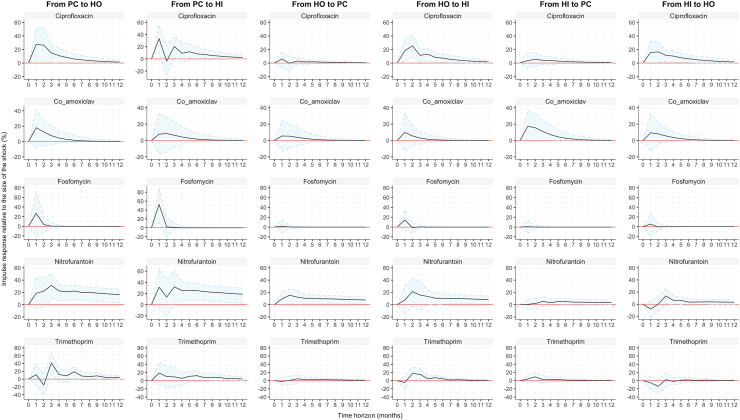
Impulse response functions of AMR in *E. coli* between primary care (PC) hospital outpatient (HO) and hospital inpatient (HI) settings. Solid lines are the estimated impulse responses of AMR after a shock consisting of a 1% relative increase of AMR in each healthcare setting, and shadows are the 95% confidence intervals determined by bootstrapping 1000 repetitions. The y-axis shows the magnitude of the response proportional to the shock. The x-axis shows the 12-month time period over which the response was traced out.

**Fig. 4:**
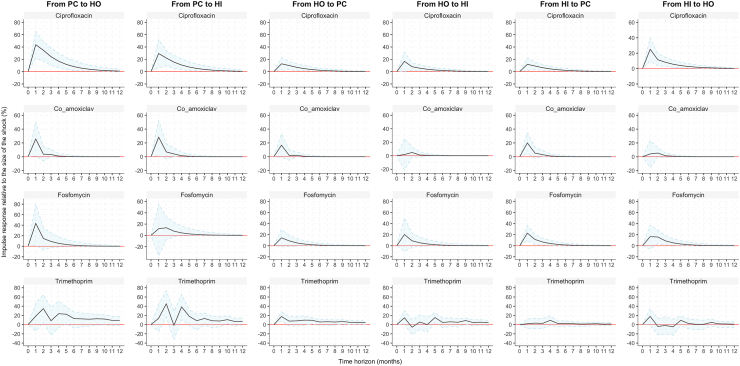
Impulse response functions of AMR in *K. pneumoniae* between primary care (PC) hospital outpatient (HO) and hospital inpatient (HI) settings. Solid lines are the estimated impulse responses of AMR after a shock consisting of a 1% relative increase of AMR in each healthcare setting, and shadows are the 95% confidence intervals determined by bootstrapping 1000 repetitions. The y-axis shows the magnitude of the response proportional to the shock. The x-axis shows the 12-month time period over which the response was traced out.

The impulse response functions, which are based on the estimated VAR models, predict that a shock (1% relative increase) to AMR in primary care would lead to a significant increase in both hospital settings, and that the magnitude of this increase would vary by bacterium–antibiotic combination. Conversely, when a hypothetical shock was applied to either hospital setting, the response was either non-significant or it was smaller compared to the response after the same shock to primary care (Fig. 3, Fig. 4).

For instance, in *E. coli*, for ciprofloxacin resistance there was an increase of approximately 0.25% (95% CI: 0.05%–0.50%) in hospital outpatients in the first month after a 1% shock to primary care, and an increase of approximately 0.25% (95% CI: 0.10%–0.51%) in hospital inpatients after a 1% shock to the hospital outpatient setting (Fig. 3). In *K. pneumoniae*, for ciprofloxacin resistance, after a 1% shock to primary care, there was an increase by approximately 0.40% (95% CI: 0.20%–0.65%) in the hospital outpatient setting, and 0.30% (95% CI: 0.10% − 0.50) in the hospital inpatient setting, peaking in the first month. A similar result was obtained when we calculated the dynamic response to a 1% shock in either hospital setting, but the size of the increase was smaller with an average of approximately 0.10% (Fig. 4).

## Discussion

This study investigated the dynamic association of AMR between primary care and hospital settings in *E. coli* and *K. pneumoniae* in the Netherlands, covering a total period of 13 years. Our findings suggest that for the majority of bacterium–antibiotic combinations, the dominant AMR association was unidirectional going from primary care to hospital settings. A notable exception was ciprofloxacin resistance, in which AMR association was bidirectional between all healthcare settings.

The dominant AMR association found in this study could be partly explained by the difference in the total amount of antibiotic use between healthcare settings. In the Netherlands, antibiotic use has been decreasing over the last decade, the majority of the total amount of antibiotic use remains at community-level with 9.73 DID (DDD/1000 inhabitant-days), compared to hospital settings where it is only 0.91 DID.^6^ Notably, the proportion of antibiotics used to treat UTIs relative to the total amount of antibiotic use is similar in primary care and in hospital settings (approximately 33%); nevertheless, the absolute numbers remain 10 times higher in primary care compared to hospitals.^6^ For some studied antibiotics, the difference in absolute amount of use is even higher. For example, nitrofurantoin is the first choice for the treatment of UTIs in primary care according to Dutch GP guidelines.^16^ Reports show a use of 1.34 DID in GP patients compared to 0.02 DID in hospital care.^25^ This absolute higher use of antibiotics in primary care could reinforce AMR development in the community, which may be eventually be transmitted to hospital settings, in line with our Granger causality results.

Previous studies found that approximately 50% of Dutch GPs follow the guidelines, and only 29% follow the guidelines when dealing with high-risk patients.^7^^,^^26^ Target interventions such as antibiotic stewardship in primary care should be considered to increase adherence to the guidelines by GPs, and they should be stimulated to take more urine cultures per patient for a better diagnosis and treatment.^7^ Antibiotic stewardship has been described as a beneficial strategy to reduce outpatient antibiotic use,^27^ and based on our findings, a decrease of AMR in primary care probably following a decrease in antibiotic use, may subsequently lead to a decrease of AMR in hospital settings.

An interesting result from our study was the bidirectional AMR association for ciprofloxacin resistance, especially in *K. pneumoniae*. Ciprofloxacin is the first-choice treatment for complicated UTIs in Dutch primary care,^16^ but it is also prescribed in outpatient hospital setting when patients do not require hospitalization and the local AMR is less than 10%.^17^ The bidirectional AMR association found in our Granger causality analysis could be explained firstly by the higher use of ciprofloxacin in primary care: on average, 0.56 DID of ciprofloxacin is used in primary care compared to 0.09 DID in hospital settings.^6^ Secondly, the use of other fluoroquinolones in both the community and in hospitals may play a role due to cross-resistance, as its usage in the community was associated with ciprofloxacin resistance in urinary bacteria.^2^^,^^4^ Moreover, co-resistance, which involves the transfer of multiple resistance genes between bacteria through plasmids,^28^ may also play a role. A study showed that the use of amoxicillin and trimethoprim was associated with ciprofloxacin resistance in urinary *E. coli.*^29^ Further studies are necessary to explore the effect of cross-resistance and co-resistance in AMR associations among healthcare settings.

We also found significant AMR associations from hospital settings to primary care for trimethoprim and fosfomycin resistance in *K. pneumoniae,* even though these antibiotics are used 10 times more frequently in primary care than in hospital settings.^6^ Theoretically, one explanation could be co-resistance to other antibiotics. Another possible explanation is that patients with complicated UTIs admitted in hospitals could become colonized by opportunist multidrug-resistant organisms (MDRO). Previous studies found that hospitals may serve as a source of MDRO, including *K. pneumoniae*, that can be further transmitted to healthcare professionals and patients.^30^^,^^31^ Thus, it could be argued that nosocomial strains may play a role in the dynamic of AMR transmission from hospital settings to primary care.

Our study has a number of strengths, but also some limitations. To our knowledge, this is the first study exploring the dynamic association of AMR between healthcare settings using multiple time series. Another study used similar methodology, but focused on the association between antibiotic use and AMR.^32^ The nature of the data used to construct the AMR time series, the careful selection of data to minimize bias in resistance percentages, and the large numbers of isolates across all Dutch healthcare settings are also strengths of this study. A possible limitation may be the risk of selection bias of isolates, especially in primary care, since Dutch GPs only culture urine in patients having either treatment failure or recurrence of UTIs.^6^ However, our calculated resistance percentages were similar to those found in all women of 11 years and older with symptoms of dysuria, strangury, or urinary frequency visiting 30 sentinel general practitioner practices of the Nivel Primary Care database.^33^ Besides, sampling guidelines, and thus the amount of selection bias, did not change during the study period, and we expect, therefore, that the calculated time trends are not influenced by the selection bias. Another limitation is that only basic epidemiological information such as age, sex and province was available in the ISIS-AR database, limiting the possibility to account for other circumstances (e.g., lifestyle), risk factors (e.g., previous use of antibiotics, reinfections, co-morbidities, hospitalizations, catheter use), UTI onset (community-acquired or hospital-acquired), and to determine differences between academic and peripheral hospitals, although the relative distribution of antibiotic use between Dutch hospitals is similar.^6^ A third limitation is that it was not possible to correct for patients going from one setting to another, because isolates of the same patient in different settings could not be linked in the ISIS-AR database. However, the statistical analysis using time series analysis does not require this knowledge.

Despite these limitations, our study contributes to the knowledge on the complexity of AMR selection and how changes of AMR in one healthcare setting may lead to changes of AMR in other healthcare settings. Our findings were obtained by applying statistical models for multiple time series that are not commonly used in health science research, but which are nevertheless powerful tools to describe these complex and dynamic systems.

In conclusion, we found that any change of AMR in primary care may predict changes of AMR in hospital settings in *E. coli* and *K. pneumoniae* for the majority of antibiotics. Notably, for ciprofloxacin resistance also any change in AMR in hospital settings may predict changes of AMR in primary care and vice versa. These results underscore the importance of antibiotic stewardship in primary care in the Netherlands as a way to prevent transmission of AMR to hospital settings. Further studies are needed to elucidate the association of AMR in different health care settings and for other antibiotics.

## Contributors

EPM contributed to conceptualization, methodology, visualization, formal analysis of the data, writing-original draft of the paper. JvR contributed to conceptualization, methodology, validation of the data analysis, and writing-review and editing of the paper. AV contributed to conceptualization, writing-review and editing of the paper. AS and the ISIS-AR study group contributed to writing-review and editing of the paper. WA contributed to data curation and writing-review and editing of the paper. All authors critically reviewed the last version of the paper and approved the final submission.

## Data sharing statement

Authors only had access to the data during the study in accordance with the license agreement signed between Erasmus Medical Center and the National Institute for Public Health and the Environment (RIVM). Data was anonymized to guarantee confidentiality of personal and health information from patients. Access to the data is subject to a signed data access agreement with RIVM.

## Declaration of interests

ISIS-AR is supported by the Dutch Ministry of Health, Welfare and Sport. EPM was supported by the Central University of Ecuador to follow a PhD program in Erasmus MC. Other authors are supported by internal funding. Authors have no conflict of interest to declare.
