# Supplementary material for: Dynamic association of antimicrobial resistance in urinary isolates of Escherichia coli and Klebsiella pneumoniae between primary care and hospital settings in the Netherlands (2008–2020): a population-based study
Source: Lancet Reg Health Eur. 2025 Jan 3;50:101197. doi: 10.1016/j.lanepe.2024.101197 (PMC11755011; doi:10.1016/j.lanepe.2024.101197)
Supplement: Supplementary Figs. S1 and S2 and Tables S1–S5 [file mmc1.docx]

**Dynamic association of antimicrobial resistance in urinary isolates of *E. coli* and *K. pneumoniae* between primary care and hospital settings in the Netherlands**

Evelyn Pamela Martínez^1,2*^, Annelies Verbon^2,3^, Annelot F. Schoffelen^4^, Wieke Altorf-van der Kuil^4^, Joost van Rosmalen^5,6,7^ on behalf of the ISIS-AR study group^‡^

^1^Facultad de Medicina Veterinaria y Zootecnia, Universidad Central del Ecuador, Quito, Ecuador;

^2^Department of Medical Microbiology and Infectious Diseases, Erasmus MC, University Medical Centre, Rotterdam, the Netherlands.

^3^Department of Infectious Diseases, Division of Internal Medicine and Dermatology, University Medical Center Utrecht, Utrecht University, the Netherlands.

^4^Centre for Infectious Diseases, Epidemiology and Surveillance, Dutch National Institute for Public Health and the Environment (RIVM), Bilthoven, The Netherlands.

^5^Department of Biostatistics, Erasmus MC, University Medical Centre, Rotterdam, the Netherlands.

^6^Department of Epidemiology, Erasmus MC, University Medical Centre, Rotterdam, the Netherlands.

^7^Julius Center for Health Sciences and Primary Care, University Medical Center Utrecht, Utrecht University, Utrecht, the Netherlands.

‡ Members of the ISIS-AR study group are listed in the Acknowledgements section.

*Corresponding author: Tel: +593(0)990785038; Fax: +593(02)2566160; E-mail: [pmartinezl@uce.edu.ec](mailto:pmartinezl@uce.edu.ec)

**Table of Contents**

[Figure S1. Map of Regions of the Netherlands. 3](#_Toc180849301)

[Table S1. Augmented Dickey-fuller (ADF) for Unit Root testing and Trend stationary testing. 4](#_Toc180849302)

[Table S2. Lag length criterion selection for VAR models according to the Akaike Information Criterion. Grey highlight values Indicates lag order selected by the criterion. 5](#_Toc180849303)

[Table S3. Specification of VAR models for *E. coli* and *K. pneumoniae*. 6](#_Toc180849304)

[Table S4. Diagnotics of VAR residuals per bacteria and antibiotic type. Diagnosis was performed to check autocorrelation, normality and equal variance. 7](#_Toc180849305)

[Figure S2. Visualization of VAR models stability with the Ordinary Least Square Cumulative Sum (OLS-CUSUM) test. 8](#_Toc180849306)

[a) VAR model for E. coli resistant to ciprofloxacin time series 8](#_Toc180849307)

[b) VAR model for E. coli resistant to Co-amoxiclav time series 9](#_Toc180849308)

[c) VAR model for E. coli resistant to Fosfomycin time series 10](#_Toc180849309)

[d) VAR model for E. coli resistant to Nitrofurantoin time series. 11](#_Toc180849310)

[e) VAR model for E. coli resistant to trimethoprim time series 12](#_Toc180849311)

[f) VAR model for K. pneumoniae resistant to ciprofloxacin time series 13](#_Toc180849312)

[g) VAR model for K. pneumoniae resistant to Co-amoxiclav time series 14](#_Toc180849313)

[h) VAR model for K. pneumoniae resistant to Fosfomycin time series 15](#_Toc180849314)

[i) VAR model for K. pneumoniae resistant to Trimethoprim time series 16](#_Toc180849315)

[Table S5. VAR model coefficients showing the dynamic association of AMR in *E. coli* and *K. pneumoniae* in the Netherlands between primary care (PC), hospital outpatient (HO) and hospital (HI). 17](#_Toc180849316)

[a) VAR model coefficients for E. coli 17](#_Toc180849317)

[b) VAR model coefficients in K. pneumoniae 19](#_Toc180849318)

# Figure S1. Map of Regions of the Netherlands.


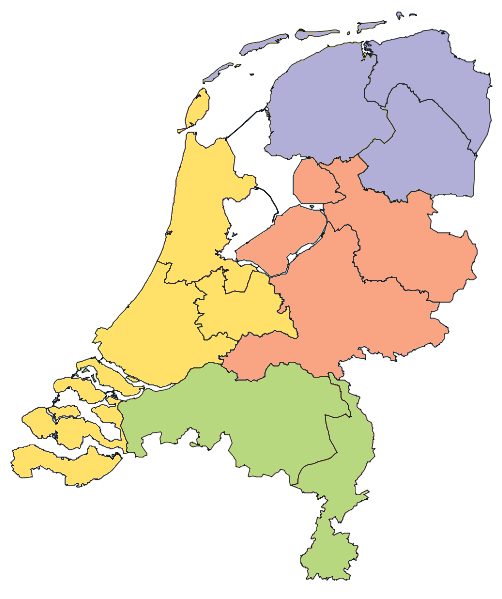


Regions were defined according to the provinces in the Netherlands: East (Orange), Overijssel, Gelderland, Flevoland; North (Purple), Groningen, Friesland, Drenthe; South (Green), Noord-Brabant, Limburg; West (Yellow), Utrecht, Noord-Holland, Zuid-Holland, Zeeland. Source: Piccolo Modificatore Laborioso, Wikimedia Commons.

# Table S1. Augmented Dickey-fuller (ADF) for Unit Root testing and Trend stationary testing.

| **Bacteria** | **Health care setting** | **Antibiotic** | **Model with drift** | | |  | **Model with drift and trend** | | |
| --- | --- | --- | --- | --- | --- | --- | --- | --- | --- |
|  |  |  | **Ho: No trend stationary** | | |  | **Ho: presence of unit root, non-stationary** | | |
|  |  |  | **Statistic** | **Critical value 5%** | **Conclusion** |  | **Statistic** | **Critical value 5%** | **Conclusion** |
| *E. coli* | Primary care | Co-amoxiclav | -5.06 | -2.89 | Trend stationary |  | -5.22 | -3.45 | Stationary |
|  | Primary care | Ciprofloxacin | -2.62 | -2.88 | No trend stationary |  | -4.88 | -3.43 | Stationary |
|  | Primary care | Fosfomycin | -5.95 | -2.89 | Trend stationary |  | -5.88 | -3.45 | Stationary |
|  | Primary care | Nitrofurantoin | -3.89 | -2.88 | Trend stationary |  | -4.89 | -3.43 | Stationary |
|  | Primary care | Trimethoprim | -1.46 | -2.88 | No trend stationary |  | -5.23 | -3.43 | Stationary |
|  | Hospital outpatient | Co-amoxiclav | -6.54 | -2.89 | Trend stationary |  | -6.51 | -3.45 | Stationary |
|  | Hospital outpatient | Ciprofloxacin | -4.93 | -2.88 | Trend stationary |  | -6.66 | -3.43 | Stationary |
|  | Hospital outpatient | Fosfomycin | -6.12 | -2.89 | Trend stationary |  | -6.47 | -3.45 | Stationary |
|  | Hospital outpatient | Nitrofurantoin | -5.19 | -2.88 | Trend stationary |  | -5.67 | -3.43 | Stationary |
|  | Hospital outpatient | Trimethoprim | -3.00 | -2.88 | No trend stationary |  | -7.07 | -3.43 | Stationary |
|  | Hospital inpatient | Co-amoxiclav | -4.29 | -2.89 | Trend stationary |  | -4.41 | -3.45 | Stationary |
|  | Hospital inpatient | Ciprofloxacin | -3.62 | -2.88 | Trend stationary |  | -5.09 | -3.43 | Stationary |
|  | Hospital inpatient | Fosfomycin | -7.25 | -2.89 | Trend stationary |  | -7.18 | -3.45 | Stationary |
|  | Hospital inpatient | Nitrofurantoin | -5.60 | -2.88 | Trend stationary |  | -5.78 | -3.43 | Stationary |
|  | Hospital inpatient | Trimethoprim | -2.47 | -2.88 | No trend stationary |  | -6.68 | -3.43 | Stationary |
|  |  |  |  |  |  |  |  |  |  |
| *K. pneumoniae* | Primary care | Co-amoxiclav | -5.92 | -2.89 | Trend stationary |  | -5.91 | -3.45 | Stationary |
|  | Primary care | Ciprofloxacin | -5.19 | -2.88 | Trend stationary |  | -5.17 | -3.43 | Stationary |
|  | Primary care | Fosfomycin | -3.69 | -2.89 | Trend stationary |  | -3.61 | -3.45 | Stationary |
|  | Primary care | Trimethoprim | -2.89 | -2.88 | No trend stationary |  | -5.50 | -3.43 | Stationary |
|  | Hospital outpatient | Co-amoxiclav | -6.02 | -2.89 | Trend stationary |  | -6.42 | -3.45 | Stationary |
|  | Hospital outpatient | Ciprofloxacin | -5.27 | -2.88 | Trend stationary |  | -5.64 | -3.43 | Stationary |
|  | Hospital outpatient | Fosfomycin | -4.73 | -2.89 | Trend stationary |  | -4.69 | -3.45 | Stationary |
|  | Hospital outpatient | Trimethoprim | -5.74 | -2.88 | Trend stationary |  | -6.37 | -3.43 | Stationary |
|  | Hospital inpatient | Co-amoxiclav | -6.28 | -2.89 | Trend stationary |  | -6.39 | -3.45 | Stationary |
|  | Hospital inpatient | Ciprofloxacin | -5.98 | -2.88 | Trend stationary |  | -6.00 | -3.43 | Stationary |
|  | Hospital inpatient | Fosfomycin | -4.62 | -2.89 | Trend stationary |  | -4.63 | -3.45 | Stationary |
|  | Hospital inpatient | Trimethoprim | -6.00 | -2.88 | Trend stationary |  | -6.60 | -3.43 | Stationary |

# Table S2. Lag length criterion selection for VAR models according to the Akaike Information Criterion. Grey highlight values Indicates lag order selected by the criterion.

| **Bacteria** | **Lag** | **Co-amoxiclav** | **Ciprofloxacin** | **Fosfomycin** | **Trimethoprim** | **Nitrofuratoin** |
| --- | --- | --- | --- | --- | --- | --- |
|  |  | **AIC(n)** | **AIC(n)** | **AIC(n)** | **AIC(n)** | **AIC(n)** |
| *E. coli* | 1 | -13.97 | -14.16 | -8.43 | -16.16 | -9.29 |
|  | 2 | -13.96 | -14.22 | -8.24 | -16.17 | -9.42 |
|  | 3 | -13.92 | -14.18 | -8.16 | -16.20 | -9.45 |
|  | 4 | -13.85 | -14.15 | -8.09 | -16.11 | -9.36 |
|  | 5 | -13.76 | -14.19 | -8.06 | -16.03 | -9.29 |
|  | 6 | -13.66 | -14.12 | -7.97 | -15.97 | -9.24 |
|  |  |  |  |  |  |  |
| *K. pneumoniae* | 1 | -7.55 | -8.25 | -10.06 | -10.39 | - |
|  | 2 | -7.44 | -8.23 | -10.01 | -10.40 | - |
|  | 3 | -7.45 | -8.21 | -9.95 | -10.34 | - |
|  | 4 | -7.42 | -8.16 | -9.88 | -10.46 | - |
|  | 5 | -7.54 | -8.15 | -9.93 | -10.39 | - |
|  | 6 | -7.39 | -8.10 | -9.77 | -10.32 | - |

# Table S3. Specification of VAR models for *E. coli* and *K. pneumoniae*.

| **N** | **Bacteria** | **Endogenous variables** | **Centered seasonal dummies** | **Lag order** | **Constant** | **Deterministic trend** |
| --- | --- | --- | --- | --- | --- | --- |
|  |  |  |  |  |  |  |
| 1 | *E. coli* | CIP-PC, CIP-HO, CIP-HI | 12 | 2 | Yes | Yes |
| 2 | *E. coli* | AMC-PC, AMC-HO, AMC-HI | 12 | 1 | Yes | No |
| 3 | *E. coli* | NIT-PC, NIT-HO, NIT-HI | 12 | 3 | Yes | No |
| 4 | *E. coli* | FOS-PC, FOS-HO, FOS-HI | 12 | 1 | Yes | No |
| 5 | *E. coli* | TMP-PC, TMP-HO, TMP-HI | 12 | 3 | Yes | Yes |
| 6 | *K. pneumoniae* | CIP-PC, CIP-HO, CIP-HI | 12 | 1 | Yes | No |
| 7 | *K. pneumoniae* | AMC-PC, AMC-HO, AMC-HI | 12 | 1 | Yes | No |
| 8 | *K. pneumoniae* | FOS-PC, FOS-HO, FOS-HI | 12 | 1 | Yes | No |
| 9 | *K. pneumoniae* | TMP-PC, TMP-HO, TMP-HI | 12 | 4 | Yes | Yes |

PC, primary care; HO, Hospital outpatient; HI, Hospital inpatient; AMC, co-amoxiclav; CIP, ciprofloxacin; FOS, fosfomycin; NIT, nitrofurantoin; TMP, trimethoprim.

# Table S4. Diagnotics of VAR residuals per bacteria and antibiotic type. Diagnosis was performed to check autocorrelation, normality and equal variance.

| **Bacteria** | **Antibiotic** | **Model type (*p*)** | **Portmanteau test** | **ARCH test** | **Jarque-Bera test** | **Skewness** | **Kurtosis** |
| --- | --- | --- | --- | --- | --- | --- | --- |
|  |  |  | **p-value** | **p-value** | **p-value** | **p-value** | **p-value** |
| *E.coli* | Ciprofloxacin | VAR(2) | 0.13 | 0.16 | 0.94 | 0.91 | 0.75 |
| *E.coli* | Co-amoxiclav | VAR(1) | 0.12 | 0.29 | 0.86 | 0.62 | 0.85 |
| *E.coli* | Fosfomycin | VAR(1) | 0.81 | 0.57 | 0.13 | 0.10 | 0.30 |
| *E.coli* | Nitrofurantoin | VAR(3) | 0.34 | 0.51 | <0.0001 | <0.0001 | 0.020 |
| *E.coli* | Trimethoprim | VAR(3) | 0.48 | 0.090 | 0.23 | 0.085 | 0.69 |
|  |  |  |  |  |  |  |  |
| *K.pneumoniae* | Ciprofloxacin | VAR(1) | 0.27 | 0.60 | <0.0001 | <0.0001 | 0.0010 |
| *K.pneumoniae* | Co-amoxiclav | VAR(1) | 0.12 | 0.29 | 0.86 | 0.62 | 0.85 |
| *K.pneumoniae* | Fosfomycin | VAR(1) | 0.09 | 0.57 | 0.80 | 0.85 | 0.51 |
| *K.pneumoniae* | Trimethoprim | VAR(4) | 0.14 | 0.95 | 0.85 | 0.54 | 0.93 |

Portmanteaus-and-Breuschi-Godfrey test for examined serial correlation of VAR residuals, Ho: non-serial correlation.

Autoregressive conditional heteroscedasticity test (ARCH) for examined equal variance of VAR, residuals, Ho: equal variance

Jarque-Bera test for examined normality of VAR residuals, Ho: normality.

Figure S2. Visualization of VAR models stability with the Ordinary Least Square Cumulative Sum (OLS-CUSUM) test. Stability is shown as a fluctuation process that should be running around the horizontal line in the middle, and never outside the 95% confidence boundary (red lines).

## a) VAR model for *E. coli* resistant to ciprofloxacin time series


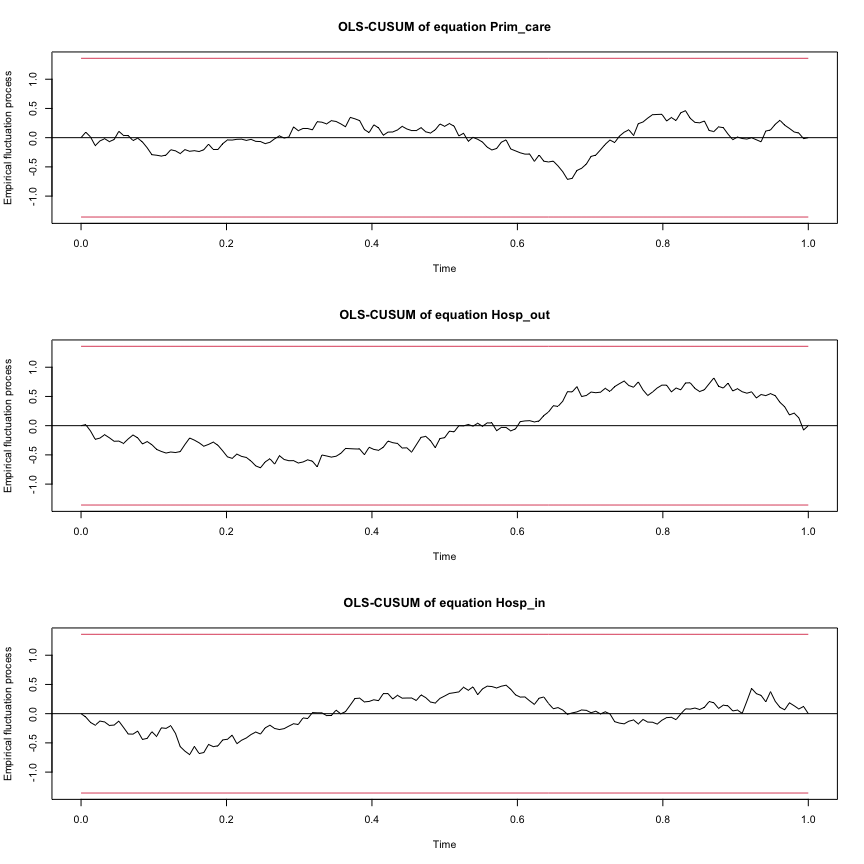


b) VAR model for *E. coli* resistant to Co-amoxiclav time series
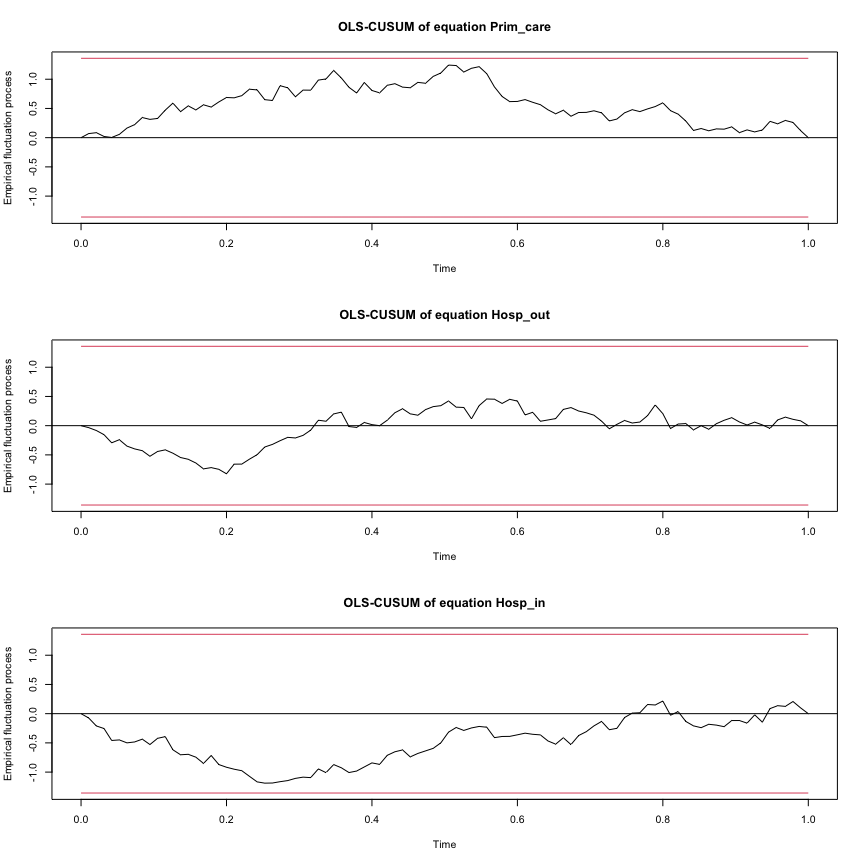


c) VAR model for *E. coli* resistant to Fosfomycin time series
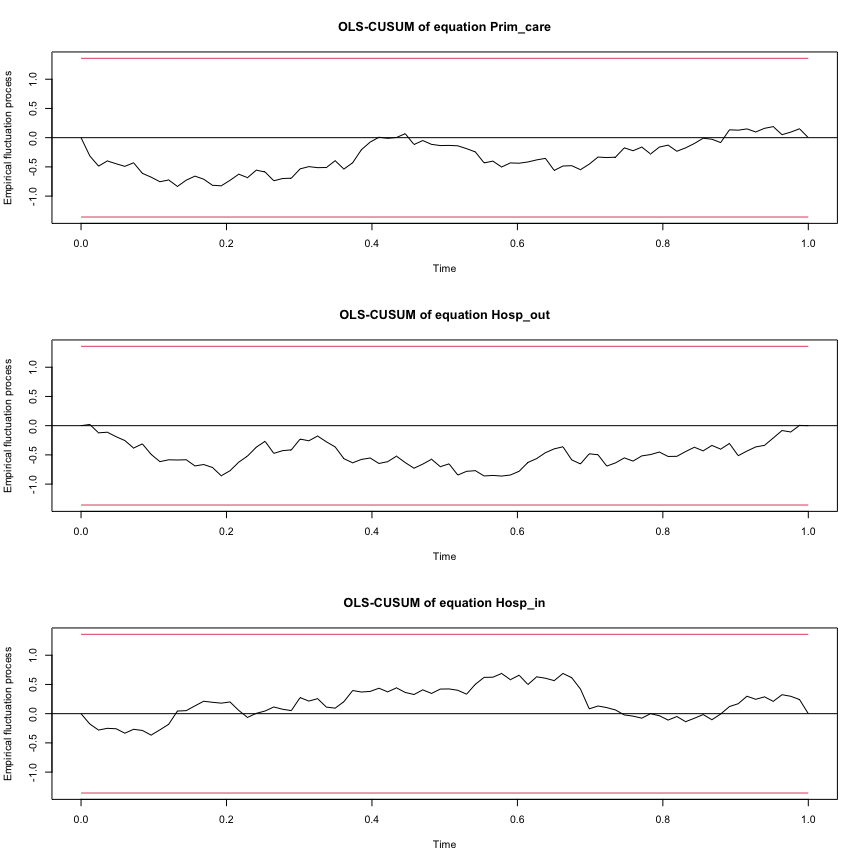


d) VAR model for *E. coli* resistant to Nitrofurantoin time series.
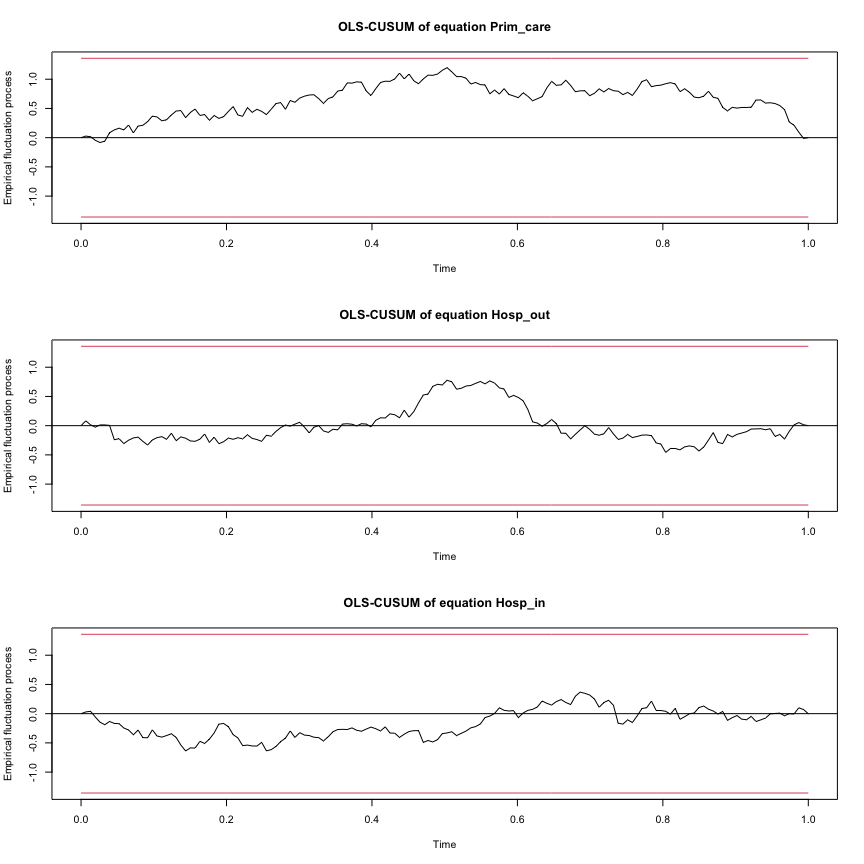


e) VAR model for *E. coli* resistant to trimethoprim time series
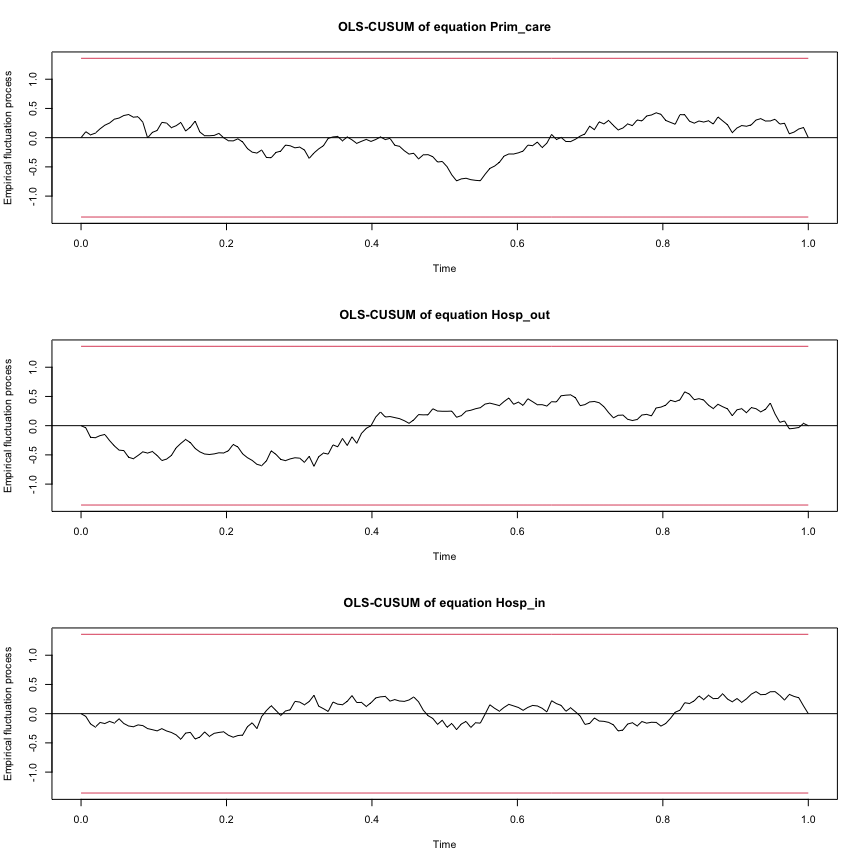


## f) VAR model for *K. pneumoniae* resistant to ciprofloxacin time series


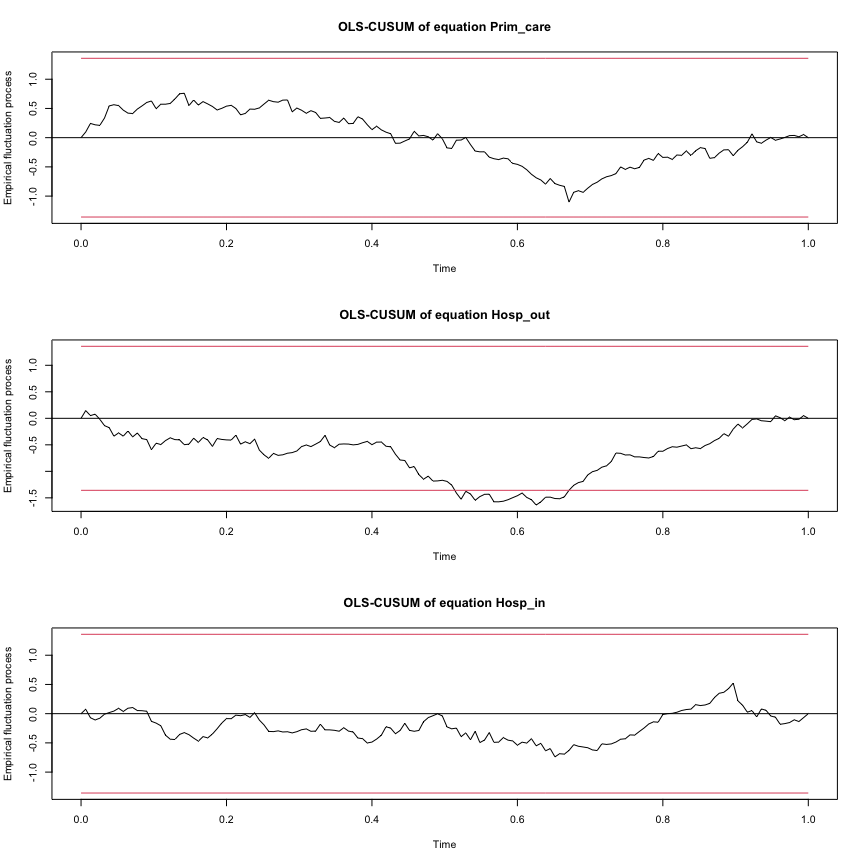


g) VAR model for *K. pneumoniae* resistant to Co-amoxiclav time series
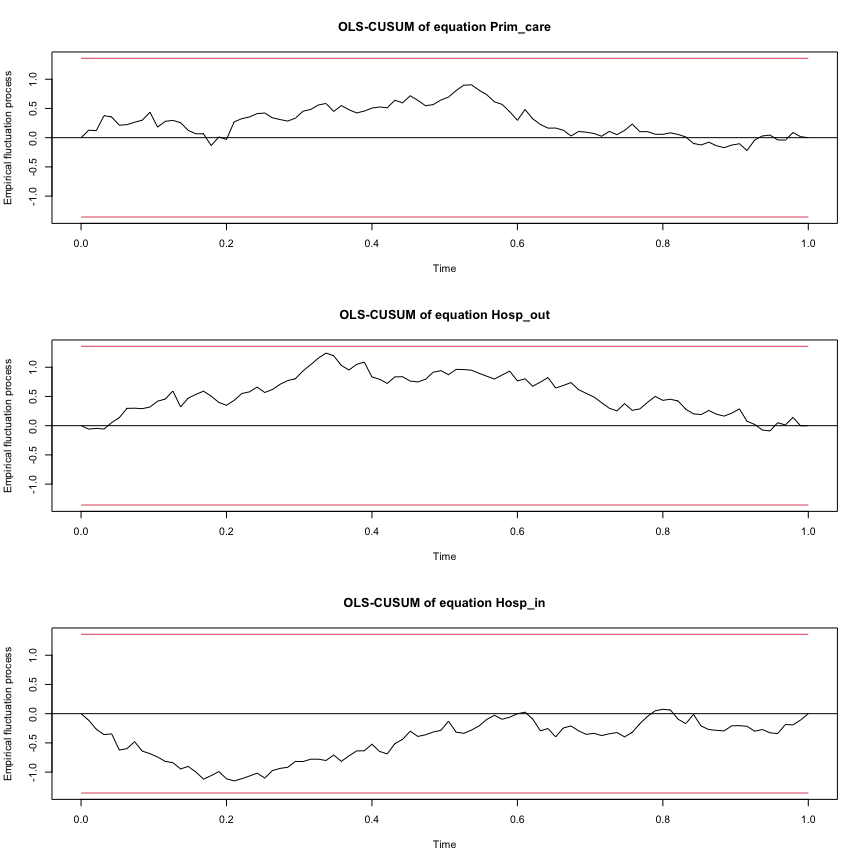


h) VAR model for *K. pneumoniae* resistant to Fosfomycin time series
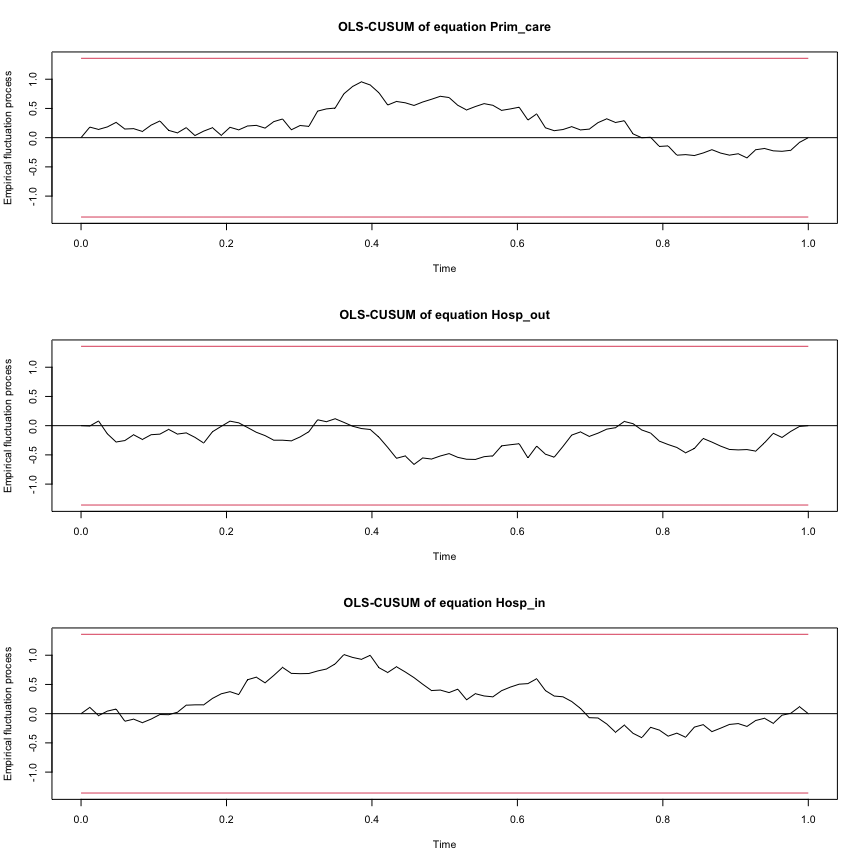


i) VAR model for *K. pneumoniae* resistant to Trimethoprim time series
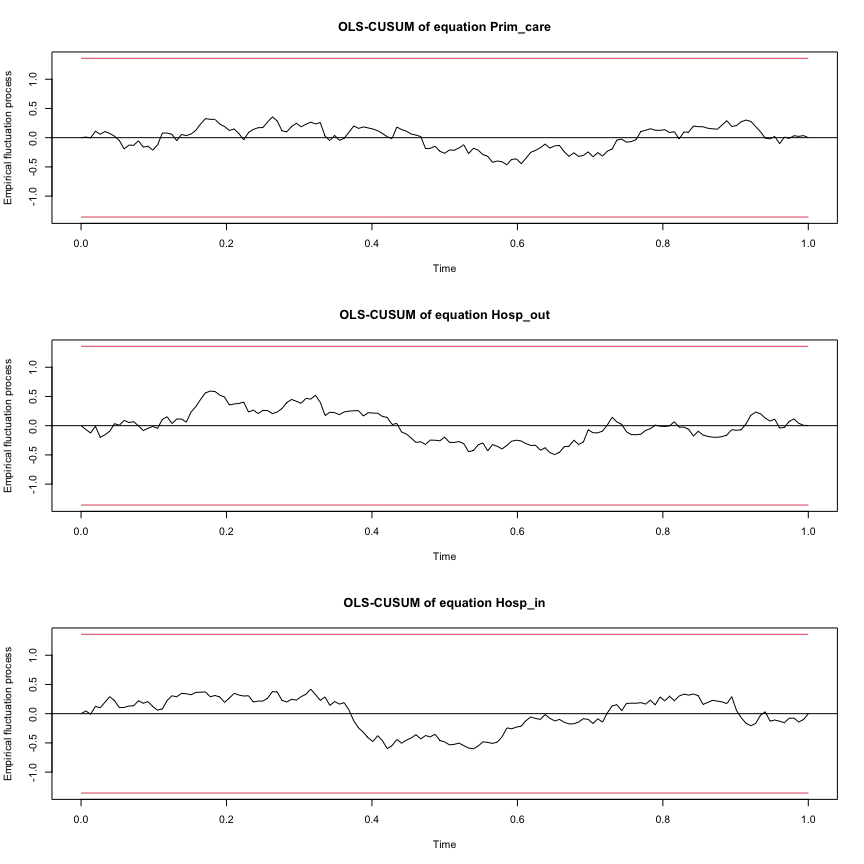


Table S5. VAR model coefficients showing the dynamic association of AMR in *E. coli* and *K. pneumoniae* in the Netherlands between primary care (PC), hospital outpatient (HO) and hospital (HI). The number of time-lag included in the models are represented by the subscript t following by a number, for intance, t-1 represent a time lag of one month.

## VAR model coefficients for *E. coli*

| **Antibiotic** | **Independent variables** | **First equation** | | | | **Second equation** | | | | **Third equation** | | | |
| --- | --- | --- | --- | --- | --- | --- | --- | --- | --- | --- | --- | --- | --- |
|  |  | **Primary care** | | | | **Hospital outpatient** | | | | **Hospital outpatient** | | | |
|  |  | **Coeff.** | **Se** | **T-test** | **P-value** | **Coeff.** | **Se** | **T-test** | **P-value** | **Coeff.** | **Se** | **T-test** | **P-value** |
| Ciprofloxacin | PC_t-1_ | 0.17 | 0.08 | 2.04 | 0.043 | 0.28 | 0.12 | 2.33 | 0.021 | 0.34 | 0.12 | 2.87 | 0.0048 |
|  | HO_t-1_ | 0.06 | 0.06 | 0.99 | 0.32 | 0.03 | 0.08 | 0.38 | 0.70 | 0.18 | 0.08 | 2.17 | 0.032 |
|  | HI_t-1_ | 0.04 | 0.06 | 0.63 | 0.53 | 0.16 | 0.08 | 1.87 | 0.063 | 0.23 | 0.08 | 2.83 | 0.0054 |
|  | PC_t-2_ | 0.17 | 0.09 | 1.86 | 0.065 | 0.15 | 0.12 | 1.25 | 0.21 | -0.23 | 0.12 | -1.84 | 0.067 |
|  | HO_t-2_ | -0.02 | 0.06 | -0.38 | 0.70 | 0.07 | 0.09 | 0.85 | 0.40 | 0.19 | 0.08 | 2.21 | 0.029 |
|  | HI_t-2_ | 0.03 | 0.06 | 0.49 | 0.63 | 0.11 | 0.08 | 1.37 | 0.17 | 0.23 | 0.08 | 2.83 | 0.0054 |
|  | Constant | 0.04 | 0.05 | 0.83 | 0.41 | 0.49 | 0.07 | 7.01 | <0.0001 | -0.11 | 0.07 | -1.62 | 0.11 |
|  | Trend | 0.00 | 0.00 | -4.67 | <0.0001 | 0.00 | 0.00 | -0.73 | 0.46 | 0.00 | 0.00 | -0.01 | 0.99 |
|  | January | 0.05 | 0.03 | 1.82 | 0.071 | 0.01 | 0.04 | 0.34 | 0.74 | 0.10 | 0.04 | 2.62 | 0.0097 |
|  | February | 0.04 | 0.03 | 1.48 | 0.14 | 0.01 | 0.04 | 0.18 | 0.86 | 0.03 | 0.04 | 0.83 | 0.41 |
|  | March | -0.01 | 0.03 | -0.22 | 0.83 | -0.08 | 0.04 | -2.08 | 0.046 | -0.01 | 0.04 | -0.36 | 0.72 |
|  | April | 0.00 | 0.03 | 0.07 | 0.95 | -0.06 | 0.04 | -1.55 | 0.12 | 0.04 | 0.04 | 1.03 | 0.31 |
|  | May | 0.00 | 0.03 | -0.17 | 0.86 | -0.03 | 0.04 | -0.86 | 0.39 | -0.04 | 0.04 | -1.00 | 0.32 |
|  | June | -0.04 | 0.03 | -1.28 | 0.20 | -0.02 | 0.04 | -0.42 | 0.67 | 0.05 | 0.04 | 1.34 | 0.18 |
|  | July | -0.05 | 0.03 | -1.74 | 0.082 | -0.07 | 0.04 | -1.90 | 0.059 | 0.05 | 0.04 | 1.30 | 0.20 |
|  | August | -0.03 | 0.03 | -1.04 | 0.30 | -0.09 | 0.04 | -2.18 | 0.031 | 0.02 | 0.04 | 0.55 | 0.58 |
|  | September | -0.02 | 0.03 | -0.82 | 0.42 | 0.02 | 0.04 | 0.46 | 0.64 | 0.01 | 0.04 | 0.32 | 0.75 |
|  | October | 0.02 | 0.03 | 0.80 | 0.42 | -0.01 | 0.04 | -0.23 | 0.82 | 0.04 | 0.04 | 0.99 | 0.32 |
|  | November | 0.00 | 0.03 | 0.14 | 0.89 | -0.01 | 0.04 | -0.38 | 0.70 | 0.03 | 0.04 | 0.76 | 0.45 |
|  | Adjusted R^2^ | 0.51 |  |  |  | 0.32 |  |  |  | 041 |  |  |  |
|  | F test | 9.16 |  |  |  | 4.93 |  |  |  | 6.82 |  |  |  |
|  | P-value | <0.0001 |  |  |  | <0.0001 |  |  |  | <0.0001 |  |  |  |
|  |  |  |  |  |  |  |  |  |  |  |  |  |  |
| Co-amoxiclav | PC_t-1_ | 0.49 | 0.10 | 4.84 | <0.0001 | 0.18 | 0.13 | 1.43 | 0.16 | 0.08 | 0.13 | 0.60 | 0.55 |
|  | HO_t-1_ | 0.06 | 0.10 | 0.58 | 0.57 | 0.16 | 0.12 | 1.36 | 0.18 | 0.10 | 0.12 | 0.81 | 0.42 |
|  | HI_t-1_ | 0.17 | 0.10 | 1.80 | 0.076 | 0.09 | 0.12 | 0.79 | 0.43 | 0.37 | 0.12 | 3.06 | 0.0031 |
|  | Constant | -0.10 | 0.02 | -4.18 | <0.0001 | 0.08 | 0.03 | 2.75 | 0.0073 | 0.06 | 0.03 | 2.16 | 0.034 |
|  | January | 0.04 | 0.04 | 0.89 | 0.38 | 0.07 | 0.05 | 1.35 | 0.18 | 0.03 | 0.05 | 0.61 | 0.54 |
|  | February | 0.05 | 0.04 | 1.28 | 0.21 | 0.09 | 0.05 | 1.76 | 0.082 | 0.09 | 0.05 | 1.70 | 0.094 |
|  | March | 0.03 | 0.04 | 0.83 | 0.41 | 0.01 | 0.05 | 0.18 | 0.86 | 0.00 | 0.05 | -0.04 | 0.97 |
|  | April | 0.01 | 0.04 | 0.13 | 0.90 | 0.04 | 0.05 | 0.80 | 0.43 | -0.01 | 0.05 | -0.14 | 0.89 |
|  | May | 0.00 | 0.04 | 0.01 | 0.99 | 0.10 | 0.05 | 1.87 | 0.065 | 0.01 | 0.05 | 0.11 | 0.91 |
|  | June | -0.02 | 0.04 | -0.58 | 0.56 | 0.05 | 0.05 | 1.02 | 0.31 | 0.00 | 0.05 | 0.02 | 0.98 |
|  | July | 0.00 | 0.04 | 0.06 | 0.95 | -0.04 | 0.05 | -0.79 | 0.43 | 0.02 | 0.05 | 0.44 | 0.66 |
|  | August | -0.01 | 0.04 | -0.12 | 0.90 | 0.00 | 0.05 | 0.01 | 0.99 | 0.08 | 0.05 | 1.54 | 0.13 |
|  | September | 0.04 | 0.04 | 1.05 | 0.30 | 0.14 | 0.05 | 2.63 | 0.010 | 0.06 | 0.05 | 1.06 | 0.29 |
|  | October | 0.04 | 0.04 | 0.86 | 0.39 | 0.00 | 0.05 | -0.01 | 0.99 | 0.08 | 0.05 | 1.56 | 0.12 |
|  | November | -0.05 | 0.04 | -1.11 | 0.27 | 0.05 | 0.05 | 0.87 | 0.39 | -0.03 | 0.05 | -0.48 | 0.63 |
|  | Adjusted R^2^ | 0.39 |  |  |  | 0.17 |  |  |  | 0.17 |  |  |  |
|  | F test | 5.24 |  |  |  | 2.38 |  |  |  | 2.40 |  |  |  |
|  | P-value | <0.0001 |  |  |  | 0.0079 |  |  |  | 0.0073 |  |  |  |
|  |  |  |  |  |  |  |  |  |  |  |  |  |  |
| Fosfomycin | PC_t-1_ | 0.08 | 0.13 | 0.65 | 0.52 | 0.27 | 0.24 | 1.14 | 0.26 | 0.53 | 0.20 | 2.58 | 0.012 |
|  | HO_t-1_ | 0.02 | 0.07 | 0.29 | 0.77 | -0.05 | 0.13 | -0.41 | 0.68 | 0.14 | 0.11 | 1.33 | 0.19 |
|  | HI_t-1_ | 0.01 | 0.07 | 0.15 | 0.88 | 0.05 | 0.13 | 0.40 | 0.69 | -0.12 | 0.11 | -1.06 | 0.29 |
|  | Constant | 0.12 | 0.03 | 3.38 | 0.0012 | 0.41 | 0.06 | 6.41 | <0.0001 | -0.06 | 0.05 | -1.02 | 0.31 |
|  | January | 0.04 | 0.09 | 0.46 | 0.65 | 0.26 | 0.17 | 1.49 | 0.14 | -0.31 | 0.15 | -2.08 | 0.041 |
|  | February | 0.00 | 0.09 | 0.05 | 0.96 | 0.29 | 0.17 | 1.65 | 0.10 | 0.04 | 0.15 | 0.27 | 0.79 |
|  | March | -0.02 | 0.09 | -0.22 | 0.83 | 0.21 | 0.16 | 1.30 | 0.20 | 0.10 | 0.14 | 0.70 | 0.49 |
|  | April | 0.07 | 0.09 | 0.78 | 0.44 | 0.21 | 0.16 | 1.29 | 0.20 | 0.10 | 0.14 | 0.69 | 0.49 |
|  | May | -0.01 | 0.09 | -0.14 | 0.89 | 0.32 | 0.17 | 1.91 | 0.060 | -0.07 | 0.14 | -0.48 | 0.63 |
|  | June | -0.03 | 0.09 | -0.30 | 0.76 | 0.24 | 0.17 | 1.44 | 0.15 | 0.21 | 0.14 | 1.44 | 0.15 |
|  | July | -0.08 | 0.09 | -0.95 | 0.35 | 0.15 | 0.16 | 0.89 | 0.37 | -0.04 | 0.14 | -0.29 | 0.78 |
|  | August | -0.12 | 0.09 | -1.35 | 0.18 | 0.11 | 0.16 | 0.69 | 0.50 | -0.02 | 0.14 | -0.15 | 0.88 |
|  | September | -0.15 | 0.09 | -1.65 | 0.10 | 0.36 | 0.16 | 2.22 | 0.030 | -0.06 | 0.14 | -0.40 | 0.69 |
|  | October | 0.01 | 0.09 | 0.09 | 0.93 | 0.18 | 0.17 | 1.08 | 0.28 | 0.01 | 0.15 | 0.10 | 0.92 |
|  | November | -0.09 | 0.09 | -0.99 | 0.33 | 0.11 | 0.17 | 0.65 | 0.52 | 0.20 | 0.14 | 1.39 | 0.17 |
|  | Adjusted R^2^ | 0.00 |  |  |  | -0.04 |  |  |  | 0.22 |  |  |  |
|  | F test | 0.99 |  |  |  | 0.78 |  |  |  | 2.68 |  |  |  |
|  | P-value | 0.47 |  |  |  | 0.69 |  |  |  | 0.0036 |  |  |  |
|  |  |  |  |  |  |  |  |  |  |  |  |  |  |
| Nitrofurantoin | PC_t-1_ | 0.15 | 0.08 | 1.76 | 0.080 | 0.18 | 0.12 | 1.51 | 0.13 | 0.31 | 0.18 | 1.79 | 0.076 |
|  | HO_t-1_ | 0.09 | 0.06 | 1.65 | 0.10 | 0.19 | 0.08 | 2.25 | 0.026 | 0.07 | 0.12 | 0.61 | 0.54 |
|  | HI_t-1_ | 0.01 | 0.04 | 0.13 | 0.89 | -0.08 | 0.06 | -1.30 | 0.20 | 0.11 | 0.09 | 1.31 | 0.19 |
|  | PC_t-2_ | 0.17 | 0.08 | 2.06 | 0.041 | 0.18 | 0.12 | 1.51 | 0.13 | 0.04 | 0.18 | 0.22 | 0.82 |
|  | HO_t-2_ | 0.12 | 0.06 | 2.12 | 0.035 | 0.09 | 0.08 | 1.03 | 0.31 | 0.16 | 0.12 | 1.33 | 0.18 |
|  | HI_t-2_ | 0.02 | 0.04 | 0.61 | 0.54 | 0.02 | 0.06 | 0.37 | 0.71 | 0.15 | 0.09 | 1.73 | 0.086 |
|  | PC_t-3_ | 0.24 | 0.08 | 2.90 | 0.0043 | 0.19 | 0.12 | 1.58 | 0.12 | 0.14 | 0.18 | 0.78 | 0.44 |
|  | HO_t-3_ | 0.05 | 0.06 | 0.80 | 0.43 | -0.06 | 0.09 | -0.66 | 0.51 | 0.03 | 0.12 | 0.23 | 0.82 |
|  | HI_t-3_ | 0.06 | 0.04 | 1.39 | 0.17 | 0.15 | 0.06 | 2.47 | 0.012 | -0.02 | 0.09 | -0.19 | 0.85 |
|  | Constant | -0.09 | 0.04 | -2.35 | 0.020 | 0.29 | 0.06 | 5.05 | <0.0001 | -0.30 | 0.08 | -3.67 | 0.00035 |
|  | January | -0.01 | 0.05 | -0.25 | 0.80 | 0.14 | 0.08 | 1.82 | 0.072 | 0.02 | 0.11 | 0.18 | 0.86 |
|  | February | -0.05 | 0.05 | -0.99 | 0.32 | 0.04 | 0.08 | 0.54 | 0.59 | 0.16 | 0.11 | 1.41 | 0.16 |
|  | March | -0.06 | 0.06 | -1.03 | 0.31 | 0.02 | 0.08 | 0.23 | 0.82 | -0.10 | 0.12 | -0.83 | 0.41 |
|  | April | 0.01 | 0.05 | 0.16 | 0.87 | -0.15 | 0.08 | -1.87 | 0.064 | -0.16 | 0.11 | -1.42 | 0.16 |
|  | May | -0.05 | 0.06 | -0.86 | 0.39 | -0.07 | 0.08 | -0.84 | 0.40 | -0.02 | 0.12 | -0.21 | 0.83 |
|  | June | -0.14 | 0.06 | -2.58 | 0.011 | -0.11 | 0.08 | -1.31 | 0.19 | -0.03 | 0.12 | -0.25 | 0.80 |
|  | July | -0.16 | 0.06 | -2.84 | 0.0152 | -0.04 | 0.08 | -0.50 | 0.62 | -0.21 | 0.12 | -1.77 | 0.079 |
|  | August | -0.18 | 0.05 | -3.35 | 0.0011 | -0.19 | 0.08 | -2.40 | 0.018 | 0.02 | 0.11 | 0.14 | 0.89 |
|  | September | -0.09 | 0.06 | -1.55 | 0.12 | 0.00 | 0.08 | -0.04 | 0.97 | -0.01 | 0.12 | -0.06 | 0.96 |
|  | October | -0.06 | 0.05 | -1.18 | 0.24 | 0.04 | 0.08 | 0.58 | 0.57 | 0.13 | 0.11 | 1.17 | 0.24 |
|  | November | -0.01 | 0.05 | -0.24 | 0.81 | 0.03 | 0.08 | 0.43 | 0.67 | -0.06 | 0.11 | -0.57 | 0.57 |
|  | Adjusted R^2^ | 0.54 |  |  |  | 0.35 |  |  |  | 0.22 |  |  |  |
|  | F test | 9.89 |  |  |  | 5.02 |  |  |  | 3.19 |  |  |  |
|  | P-value | <0.0001 |  |  |  | <0.0001 |  |  |  | <0.0001 |  |  |  |
|  |  |  |  |  |  |  |  |  |  |  |  |  |  |
|  |  |  |  |  |  |  |  |  |  |  |  |  |  |
| Trimethoprim | PC_t-1_ | 0.25 | 0.09 | 2.78 | 0.0062 | 0.11 | 0.14 | 0.78 | 0.44 | 0.19 | 0.14 | 1.35 | 0.18 |
|  | HO_t-1_ | -0.02 | 0.05 | -0.37 | 0.71 | 0.04 | 0.09 | 0.51 | 0.61 | -0.04 | 0.08 | -0.55 | 0.58 |
|  | HI_t-1_ | 0.04 | 0.06 | 0.74 | 0.46 | -0.05 | 0.09 | -0.59 | 0.56 | 0.14 | 0.09 | 1.67 | 0.10 |
|  | PC_t-2_ | 0.15 | 0.09 | 1.70 | 0.092 | -0.17 | 0.15 | -1.19 | 0.24 | 0.03 | 0.14 | 0.23 | 0.82 |
|  | HO_t-2_ | 0.01 | 0.05 | 0.25 | 0.80 | 0.11 | 0.08 | 1.30 | 0.20 | 0.19 | 0.08 | 2.36 | 0.019 |
|  | HI_t-2_ | 0.08 | 0.06 | 1.36 | 0.18 | -0.13 | 0.09 | -1.44 | 0.15 | 0.00 | 0.09 | 0.04 | 0.97 |
|  | PC_t-3_ | 0.18 | 0.09 | 2.01 | 0.046 | 0.46 | 0.14 | 3.16 | 0.0019 | 0.00 | 0.14 | 0.00 | 0.99 |
|  | HO_t-3_ | 0.04 | 0.05 | 0.85 | 0.40 | 0.14 | 0.08 | 1.68 | 0.095 | 0.13 | 0.08 | 1.56 | 0.12 |
|  | HI_t-3_ | -0.02 | 0.06 | -0.35 | 0.73 | 0.05 | 0.09 | 0.57 | 0.57 | 0.08 | 0.09 | 0.89 | 0.38 |
|  | Constant | -0.03 | 0.02 | -1.76 | 0.080 | 0.10 | 0.03 | 4.05 | <0.0001 | -0.04 | 0.02 | -1.72 | 0.087 |
|  | Trend | 0.00 | 0.00 | -2.90 | 0.0044 | 0.00 | 0.00 | -1.50 | 0.14 | 0.00 | 0.00 | -1.29 | 0.20 |
|  | January | 0.06 | 0.02 | 2.99 | 0.0033 | -0.04 | 0.03 | -1.27 | 0.21 | 0.01 | 0.03 | 0.20 | 0.84 |
|  | February | 0.00 | 0.02 | -0.17 | 0.86 | -0.04 | 0.03 | -1.20 | 0.23 | -0.01 | 0.03 | -0.35 | 0.73 |
|  | March | -0.01 | 0.02 | -0.43 | 0.67 | -0.07 | 0.03 | -2.18 | 0.031 | -0.02 | 0.03 | -0.58 | 0.56 |
|  | April | -0.03 | 0.02 | -1.59 | 0.11 | -0.10 | 0.03 | -3.12 | 0.0022 | -0.04 | 0.03 | -1.36 | 0.18 |
|  | May | -0.03 | 0.02 | -1.45 | 0.15 | -0.09 | 0.03 | -2.77 | 0.0064 | -0.08 | 0.03 | -2.67 | 0.0085 |
|  | June | -0.04 | 0.02 | -1.83 | 0.069 | -0.14 | 0.03 | -4.38 | <0.0001 | -0.01 | 0.03 | -0.25 | 0.80 |
|  | July | -0.04 | 0.02 | -1.91 | 0.058 | -0.11 | 0.03 | -3.62 | 0.00042 | -0.01 | 0.03 | -0.36 | 0.72 |
|  | August | -0.05 | 0.02 | -2.47 | 0.015 | -0.10 | 0.03 | -3.09 | 0.0025 | -0.02 | 0.03 | -0.63 | 0.53 |
|  | September | -0.01 | 0.02 | -0.48 | 0.64 | -0.05 | 0.03 | -1.57 | 0.12 | -0.03 | 0.03 | -0.82 | 0.41 |
|  | October | 0.03 | 0.02 | 1.37 | 0.17 | -0.03 | 0.03 | -1.08 | 0.28 | 0.01 | 0.03 | 0.24 | 0.81 |
|  | November | 0.01 | 0.02 | 0.61 | 0.55 | -0.06 | 0.03 | -2.03 | 0.044 | -0.03 | 0.03 | -0.89 | 0.38 |
|  | Adjusted R^2^ | 0.91 |  |  |  | 0.69 |  |  |  | 0.72 |  |  |  |
|  | F test | 69.91 |  |  |  | 17.14 |  |  |  | 19.91 |  |  |  |
|  | P-value | <0.0001 |  |  |  | <0.0001 |  |  |  | <0.0001 |  |  |  |

## VAR model coefficients in *K. pneumoniae*

| **Antibiotic** | **Independent variables** | **First equation** | | | | **Second equation** | | | | **Third equation** | | | |
| --- | --- | --- | --- | --- | --- | --- | --- | --- | --- | --- | --- | --- | --- |
|  |  | **Primary care** | | | | **Hospital outpatient** | | | | **Hospital outpatient** | | | |
|  |  | **Coeff.** | **Se** | **T-test** | **P-value** | **Coeff.** | **Se** | **T-test** | **P-value** | **Coeff.** | **Se** | **T-test** | **P-value** |
| Ciprofloxacin | PC_t-1_ | 0.44 | 0.07 | 6.02 | <0.0001 | 0.44 | 0.11 | 3.99 | 0.00011 | 0.29 | 0.12 | 2.53 | 0.013 |
|  | HO_t-1_ | 0.13 | 0.05 | 2.47 | 0.015 | 0.19 | 0.08 | 2.38 | 0.019 | 0.17 | 0.08 | 2.02 | 0.045 |
|  | HI_t-1_ | 0.12 | 0.05 | 2.27 | 0.02 | 0.25 | 0.08 | 3.17 | 0.0019 | 0.06 | 0.08 | 0.76 | 0.45 |
|  | Constant | 0.00 | 0.02 | -0.10 | 0.92 | 0.07 | 0.03 | 2.73 | 0.0072 | -0.17 | 0.03 | -5.91 | <0.0001 |
|  | January | -0.07 | 0.07 | -1.00 | 0.32 | -0.05 | 0.11 | -0.47 | 0.64 | -0.05 | 0.12 | -0.47 | 0.64 |
|  | February | -0.01 | 0.07 | -0.16 | 0.88 | 0.00 | 0.11 | 0.03 | 0.97 | -0.17 | 0.11 | -1.50 | 0.14 |
|  | March | -0.06 | 0.07 | -0.90 | 0.37 | 0.00 | 0.11 | -0.03 | 0.98 | -0.15 | 0.11 | -1.30 | 0.19 |
|  | April | -0.13 | 0.07 | -1.80 | 0.075 | -0.05 | 0.11 | -0.50 | 0.62 | -0.12 | 0.11 | -1.11 | 0.27 |
|  | May | -0.09 | 0.07 | -1.24 | 0.22 | -0.21 | 0.11 | -2.01 | 0.047 | -0.09 | 0.11 | -0.76 | 0.45 |
|  | June | -0.01 | 0.07 | -0.12 | 0.91 | 0.02 | 0.11 | 0.17 | 0.87 | 0.07 | 0.11 | 0.62 | 0.54 |
|  | July | -0.11 | 0.07 | -1.50 | 0.14 | -0.07 | 0.11 | -0.70 | 0.48 | -0.08 | 0.11 | -0.67 | 0.50 |
|  | August | -0.15 | 0.07 | -2.10 | 0.04 | -0.03 | 0.11 | -0.29 | 0.77 | -0.06 | 0.11 | -0.55 | 0.58 |
|  | September | 0.02 | 0.07 | 0.22 | 0.82 | -0.07 | 0.11 | -0.64 | 0.52 | 0.05 | 0.11 | 0.40 | 0.69 |
|  | October | -0.05 | 0.07 | -0.64 | 0.52 | 0.02 | 0.11 | 0.16 | 0.88 | -0.04 | 0.11 | -0.32 | 0.75 |
|  | November | 0.00 | 0.07 | -0.07 | 0.95 | 0.01 | 0.11 | 0.07 | 0.94 | -0.05 | 0.11 | -0.47 | 0.64 |
|  | Adjusted R^2^ | 0.35 |  |  |  | 0.27 |  |  |  | 0.07 |  |  |  |
|  | F test | 6.90 |  |  |  | 5.08 |  |  |  | 1.80 |  |  |  |
|  | P-value | <0.0001 |  |  |  | <0.0001 |  |  |  | 0.045 |  |  |  |
|  |  |  |  |  |  |  |  |  |  |  |  |  |  |
|  |  |  |  |  |  |  |  |  |  |  |  |  |  |
| Co-amoxiclav | PC_t-1_ | 0.14 | 0.10 | 1.38 | 0.17 | 0.26 | 0.13 | 2.04 | 0.045 | 0.28 | 0.13 | 2.12 | 0.037 |
|  | HO_t-1_ | 0.17 | 0.09 | 1.92 | 0.058 | -0.04 | 0.11 | -0.36 | 0.72 | 0.02 | 0.12 | 0.15 | 0.88 |
|  | HI_t-1_ | 0.20 | 0.08 | 2.44 | 0.017 | 0.04 | 0.10 | 0.40 | 0.69 | 0.09 | 0.11 | 0.83 | 0.41 |
|  | Constant | -0.40 | 0.08 | -4.96 | <0.0001 | -0.35 | 0.10 | -3.48 | 0.00081 | -0.12 | 0.11 | -1.15 | 0.25 |
|  | January | -0.12 | 0.12 | -1.00 | 0.32 | 0.04 | 0.15 | 0.24 | 0.81 | 0.08 | 0.16 | 0.51 | 0.61 |
|  | February | -0.12 | 0.12 | -0.97 | 0.34 | 0.01 | 0.15 | 0.08 | 0.93 | -0.02 | 0.16 | -0.14 | 0.89 |
|  | March | -0.02 | 0.12 | -0.19 | 0.85 | 0.16 | 0.15 | 1.09 | 0.28 | -0.23 | 0.16 | -1.50 | 0.14 |
|  | April | -0.27 | 0.12 | -2.33 | 0.023 | -0.01 | 0.15 | -0.05 | 0.96 | 0.00 | 0.15 | -0.03 | 0.97 |
|  | May | -0.02 | 0.12 | -0.20 | 0.84 | 0.08 | 0.15 | 0.54 | 0.59 | -0.01 | 0.16 | -0.07 | 0.94 |
|  | June | -0.12 | 0.12 | -0.99 | 0.32 | 0.11 | 0.15 | 0.75 | 0.46 | -0.22 | 0.16 | -1.39 | 0.17 |
|  | July | -0.11 | 0.12 | -0.99 | 0.32 | 0.21 | 0.14 | 1.48 | 0.14 | -0.07 | 0.15 | -0.49 | 0.63 |
|  | August | -0.14 | 0.12 | -1.23 | 0.22 | 0.11 | 0.15 | 0.73 | 0.47 | -0.04 | 0.15 | -0.27 | 0.79 |
|  | September | -0.10 | 0.12 | -0.87 | 0.39 | 0.26 | 0.15 | 1.77 | 0.08 | -0.03 | 0.15 | -0.22 | 0.82 |
|  | October | -0.11 | 0.12 | -0.92 | 0.36 | 0.28 | 0.15 | 1.93 | 0.057 | -0.06 | 0.16 | -0.38 | 0.71 |
|  | November | -0.16 | 0.12 | -1.37 | 0.18 | 0.15 | 0.15 | 1.02 | 0.31 | -0.28 | 0.16 | -1.78 | 0.08 |
|  | Adjusted R^2^ | 0.07 |  |  |  | 0.00 |  |  |  | 0.02 |  |  |  |
|  | F test | 1.48 |  |  |  | 1.00 |  |  |  | 1.12 |  |  |  |
|  | P-value | 0.14 |  |  |  | 0.46 |  |  |  | 0.36 |  |  |  |
|  |  |  |  |  |  |  |  |  |  |  |  |  |  |
| Fosfomycin | PC_t-1_ | 0.17 | 0.13 | 1.26 | 0.21 | 0.44 | 0.20 | 2.15 | 0.035 | 0.12 | 0.25 | 0.47 | 0.64 |
|  | HO_t-1_ | 0.14 | 0.08 | 1.82 | 0.073 | 0.13 | 0.12 | 1.05 | 0.30 | 0.20 | 0.15 | 1.35 | 0.18 |
|  | HI_t-1_ | 0.23 | 0.07 | 3.25 | 0.0018 | 0.16 | 0.11 | 1.51 | 0.14 | 0.23 | 0.13 | 1.70 | 0.094 |
|  | Constant | 0.12 | 0.03 | 4.14 | <0.0001 | 0.06 | 0.05 | 1.30 | 0.20 | -0.25 | 0.06 | -4.39 | <0.0001 |
|  | January | 0.14 | 0.07 | 1.83 | 0.072 | 0.06 | 0.11 | 0.54 | 0.59 | -0.13 | 0.14 | -0.89 | 0.38 |
|  | February | 0.05 | 0.07 | 0.70 | 0.49 | 0.06 | 0.11 | 0.57 | 0.57 | 0.12 | 0.13 | 0.93 | 0.36 |
|  | March | 0.09 | 0.07 | 1.24 | 0.22 | -0.19 | 0.11 | -1.75 | 0.084 | 0.03 | 0.14 | 0.21 | 0.83 |
|  | April | 0.04 | 0.07 | 0.60 | 0.55 | 0.06 | 0.11 | 0.55 | 0.59 | 0.06 | 0.14 | 0.42 | 0.67 |
|  | May | 0.04 | 0.07 | 0.61 | 0.54 | -0.01 | 0.11 | -0.12 | 0.91 | 0.07 | 0.13 | 0.52 | 0.60 |
|  | June | 0.06 | 0.07 | 0.87 | 0.39 | 0.02 | 0.11 | 0.19 | 0.85 | -0.15 | 0.13 | -1.15 | 0.25 |
|  | July | 0.03 | 0.07 | 0.37 | 0.71 | 0.06 | 0.11 | 0.60 | 0.55 | 0.01 | 0.13 | 0.06 | 0.95 |
|  | August | -0.06 | 0.07 | -0.88 | 0.38 | 0.03 | 0.11 | 0.27 | 0.79 | -0.01 | 0.13 | -0.11 | 0.91 |
|  | September | -0.02 | 0.07 | -0.33 | 0.74 | -0.03 | 0.11 | -0.24 | 0.82 | -0.07 | 0.14 | -0.50 | 0.62 |
|  | October | 0.05 | 0.07 | 0.74 | 0.46 | 0.07 | 0.11 | 0.65 | 0.52 | -0.25 | 0.13 | -1.84 | 0.069 |
|  | November | 0.10 | 0.07 | 1.40 | 0.16 | 0.01 | 0.11 | 0.06 | 0.95 | -0.07 | 0.13 | -0.53 | 0.60 |
|  | Adjusted R^2^ | 0.32 |  |  |  | 0.19 |  |  |  | 0.15 |  |  |  |
|  | F test | 3.71 |  |  |  | 2.37 |  |  |  | 2.01 |  |  |  |
|  | P-value | 0.00013 |  |  |  | 0.0095 |  |  |  | 0.029 |  |  |  |
|  |  |  |  |  |  |  |  |  |  |  |  |  |  |
| Trimethoprim | PC_t-1_ | 0.18 | 0.09 | 2.06 | 0.041 | 0.19 | 0.15 | 1.22 | 0.22 | 0.14 | 0.15 | 0.95 | 0.35 |
|  | HO_t-1_ | 0.18 | 0.05 | 3.58 | 0.00049 | 0.08 | 0.08 | 0.96 | 0.34 | 0.15 | 0.08 | 1.86 | 0.065 |
|  | HI_t-1_ | 0.02 | 0.05 | 0.35 | 0.72 | 0.18 | 0.09 | 2.00 | 0.048 | 0.06 | 0.09 | 0.73 | 0.47 |
|  | PC_t-2_ | 0.05 | 0.09 | 0.58 | 0.57 | 0.27 | 0.15 | 1.77 | 0.079 | 0.39 | 0.15 | 2.62 | 0.0099 |
|  | HO_t-2_ | 0.03 | 0.05 | 0.52 | 0.60 | -0.06 | 0.09 | -0.71 | 0.48 | -0.10 | 0.09 | -1.21 | 0.23 |
|  | HI_t-2_ | 0.00 | 0.05 | -0.06 | 0.95 | -0.07 | 0.09 | -0.75 | 0.45 | 0.03 | 0.09 | 0.38 | 0.71 |
|  | PC_t-3_ | 0.09 | 0.09 | 0.92 | 0.36 | -0.08 | 0.16 | -0.48 | 0.63 | -0.17 | 0.15 | -1.12 | 0.26 |
|  | HO_t-3_ | 0.06 | 0.05 | 1.10 | 0.27 | 0.04 | 0.09 | 0.50 | 0.62 | -0.02 | 0.09 | -0.19 | 0.85 |
|  | HI_t-3_ | 0.02 | 0.05 | 0.39 | 0.70 | -0.02 | 0.09 | -0.27 | 0.79 | 0.02 | 0.09 | 0.25 | 0.80 |
|  | PC_t-4_ | -0.09 | 0.09 | -1.05 | 0.29 | 0.23 | 0.15 | 1.49 | 0.14 | 0.35 | 0.15 | 2.40 | 0.0178 |
|  | HO_t-4_ | 0.04 | 0.05 | 0.79 | 0.43 | 0.33 | 0.09 | 3.82 | 0.00021 | -0.03 | 0.08 | -0.30 | 0.76 |
|  | HI_t-4_ | 0.08 | 0.05 | 1.54 | 0.13 | -0.06 | 0.09 | -0.75 | 0.45 | 0.09 | 0.08 | 1.02 | 0.31 |
|  | Constant | 0.07 | 0.05 | 1.32 | 0.19 | -0.09 | 0.09 | -1.10 | 0.28 | -0.39 | 0.08 | -4.63 | <0.0001 |
|  | Trend | 0.00 | 0.00 | -4.57 | <0.0001 | 0.00 | 0.00 | 1.51 | 0.13 | 0.00 | 0.00 | 2.05 | 0.04 |
|  | January | -0.10 | 0.05 | -2.09 | 0.038 | 0.04 | 0.08 | 0.46 | 0.65 | -0.04 | 0.08 | -0.46 | 0.65 |
|  | February | -0.10 | 0.05 | -1.99 | 0.049 | -0.08 | 0.08 | -0.91 | 0.36 | -0.22 | 0.08 | -2.76 | 0.0066 |
|  | March | -0.11 | 0.05 | -2.29 | 0.023 | -0.02 | 0.08 | -0.24 | 0.81 | -0.15 | 0.08 | -1.78 | 0.077 |
|  | April | -0.09 | 0.05 | -1.86 | 0.065 | -0.05 | 0.09 | -0.59 | 0.56 | -0.19 | 0.08 | -2.28 | 0.024 |
|  | May | -0.08 | 0.05 | -1.69 | 0.093 | -0.20 | 0.08 | -2.45 | 0.0156 | 0.01 | 0.08 | 0.16 | 0.88 |
|  | June | -0.05 | 0.05 | -1.10 | 0.28 | -0.14 | 0.08 | -1.70 | 0.092 | -0.08 | 0.08 | -1.04 | 0.30 |
|  | July | -0.12 | 0.05 | -2.52 | 0.013 | -0.02 | 0.08 | -0.20 | 0.84 | -0.06 | 0.08 | -0.69 | 0.49 |
|  | August | -0.15 | 0.05 | -2.99 | 0.0034 | 0.05 | 0.08 | 0.57 | 0.57 | -0.06 | 0.08 | -0.77 | 0.44 |
|  | September | -0.08 | 0.05 | -1.65 | 0.10 | 0.05 | 0.08 | 0.57 | 0.57 | -0.11 | 0.08 | -1.31 | 0.19 |
|  | October | -0.08 | 0.05 | -1.75 | 0.08 | 0.03 | 0.08 | 0.42 | 0.67 | -0.15 | 0.08 | -1.93 | 0.055 |
|  | November | -0.04 | 0.05 | -0.96 | 0.34 | 0.09 | 0.08 | 1.09 | 0.28 | -0.10 | 0.08 | -1.27 | 0.21 |
|  | Adjusted R^2^ | 0.77 |  |  |  | 0.47 |  |  |  | 0.27 |  |  |  |
|  | F test | 21.26 |  |  |  | 4.68 |  |  |  | 3.33 |  |  |  |
|  | P-value | <0.0001 |  |  |  | <0.0001 |  |  |  | <0.0001 |  |  |  |
